# Leaf reflectance can surrogate foliar economics better than physiological traits across macrophyte species

**DOI:** 10.1186/s13007-021-00816-4

**Published:** 2021-11-10

**Authors:** Paolo Villa, Rossano Bolpagni, Monica Pinardi, Viktor R. Tóth

**Affiliations:** 1 Institute for Electromagnetic Sensing of the Environment, National Research Council of Italy (CNR-IREA), Milan, Italy; 2 Department of Chemistry, Life Sciences and Environmental Sustainability, University of Parma, Parma, Italy; 3 Balaton Limnological Research Institute, Tihany, Hungary

**Keywords:** Aquatic plants, Functional traits, Intraspecific variability, Leaf economics spectrum (LES), Remote sensing, Spectroscopy

## Abstract

**Background:**

Macrophytes are key players in aquatic ecosystems diversity, but knowledge on variability of their functional traits, among and within species, is still limited. Remote sensing is a high-throughput, feasible option for characterizing plant traits at different scales, provided that reliable spectroscopy models are calibrated with congruous empirical data, but existing applications are biased towards terrestrial plants. We sampled leaves from six floating and emergent macrophyte species common in temperate areas, covering different phenological stages, seasons, and environmental conditions, and measured leaf reflectance (400–2500 nm) and leaf traits (dealing with photophysiology, pigments, and structure). We explored optimal spectral band combinations and established non-parametric reflectance-based models for selected traits, eventually showing how airborne hyperspectral data could capture spatial–temporal macrophyte variability.

**Results:**

Our key finding is that structural—leaf dry matter content, leaf mass per area—and biochemical—chlorophyll-a content and chlorophylls to carotenoids ratio—traits can be surrogated by leaf reflectance with normalized error under 17% across macrophyte species. On the other hand, the performance of reflectance-based models for photophysiological traits substantively varies, depending on macrophyte species and target parameters.

**Conclusions:**

Our main results show the link between leaf reflectance and leaf economics (structure and biochemistry) for aquatic plants, thus envisioning a crucial role for remote sensing in enhancing the level of detail of macrophyte functional diversity analysis to intra-site and intra-species scales. At the same time, we highlighted some difficulties in establishing a general link between reflectance and photosynthetic performance under high environmental heterogeneity, potentially opening further investigation directions.

**Supplementary Information:**

The online version contains supplementary material available at 10.1186/s13007-021-00816-4.

## Background

At the borders of land and water, incorporating both aquatic and terrestrial features, wetlands are among the most important [1, 2], most productive [3] and most diverse [4] ecosystems in temperate areas. The origin of this diversity is attributed both to their transitional status [5] and to the high spatial and temporal variability of environmental conditions [6]. Macrovegetation of these areas is acclimated to the seasonal and spatial changes of their habitat, created along the major environmental gradient transition from one ecotype to another in monospecific stands, and coenoclines in mixed stands [7, 8]. Aquatic plants are key ecosystem players in littoral ecotones, as they are hotspots of biogeochemical cycling, actively impact the ecosystem by regulating water flow and sedimentation, and promote biodiversity by attracting and sheltering a large number of species ([9–12]. At the same time, wetlands diversity and functioning are dramatically impacted by alien plants invasions [13].

Environmental heterogeneity, in joint action with genetic variability and morpho-functional plasticity of macrophyte species, result in faceted phenotypic and phenological adaptations and/or acclimations within the local populations, shaping communities and even individuals [7, 14–19]. Multi-dimensional trait variability is a recent object of investigation in functional ecology [20–22]. Knowledge about ranges and interconnections of trait variability within aquatic plant communities (populations) is still relatively limited, probably due to the peculiar features of macrophytes and the environmental patchiness of their habitat [8]. The issue of disentangling the effects of inter-specific and intra-specific trait variability is at the centre of the current debate [23–25], especially for key traits related to the leaf economics spectrum (LES, [26]). Moreover, investigating spatial patterns of trait variability at local scales is a relatively recent topic [27–30] and some light has still to be shed on this level of heterogeneity in plant communities, with implications on productivity and connected processes [31].

Exploring this fine-scale variability with direct measurements, usually carried out in situ and in the laboratory, is very time and resource consuming, and often logistically constrained in aquatic systems. The level of maturity in platforms and techniques achieved by remote sensing (RS) make it a feasible and potentially very effective way forward in characterizing selected plant trait variability within communities at different geographic scales [32–34], overcoming logistic and economic constraints.

In particular, the last two decades have seen the development of a range of applications for RS of plant bio-physical and bio-chemical traits, with an intensification of this trend in the last decade [35–37]. RS-based works have by a large majority focused on terrestrial plants, from forest and grassland biomes (e.g. [32, 38–43], but some developments on wetland and aquatic vegetation have been recently documented [44–46]. As aquatic plants feature significant differences with respect to terrestrial ones in terms of morphology and physiology ([47–49], the relations between leaf optical properties and reflectance at the basis of functional traits modelling of terrestrial vegetation, such as the ones embedded into PROSPECT models [50], cannot be taken by granted, and to our best knowledge, a systematic check of those relations in current literature is still lacking. For being effective, applications of RS for mapping plant functional traits require an analysis of which traits can be modelled from spectral reflectance, and this is preferably done using data covering natural trait heterogeneity. Empirical approaches for assessing reflectance spectra as a proxy of plant traits, at leaf or canopy level, normally employ spectroscopy-based methods ranging from parametric regression models input with spectral indices, computed as band combinations (e.g. [51–53]) to non-parametric regression models, such as partial least-square regression (e.g. [38, 54–56]).

Towards meaningful applications of RS for plant functional ecology, the fundamental question is: which leaf traits, within a specific plant group, can be reliably modelled (and which others cannot) using spectroscopy? With this study, we aim to provide an answer to this question—which has been already explored over a range of terrestrial species—for what concerns floating and emergent aquatic plants common in temperate areas. To tackle this question, we purposely collected empirical data of leaf spectra and a set of leaf traits—dealing with photophysiology, pigments and leaf structure—from six macrophyte species under different times, seasons and environmental conditions, over three sites located in Europe.

The objectives of this work are: (i) to assess the variability in leaf functional traits within and among macrophyte species, with attention to alien vs. native taxa dualism; (ii) to evaluate which functional traits—photophysiological, biochemical and structural—can be effectively modelled across macrophyte species using leaf reflectance, and which wavelength ranges and combinations are more sensitive to specific traits; and (iii) to test how spectral proxies for selected traits can be exploited using remote sensing data for the straightforward visualization of spatial and temporal functional variability of macrophyte communities, at inter- and intra-species level.

## Methods

### Sampling site descriptions

Macrophyte samples were collected from three temperate shallow lakes surrounded by wetlands and hosting abundant macrophyte communities, located in central and southern Europe: Lake Hídvégi (Hungary), Mantua lakes system (Italy), and Lake Varese (Italy).

Lake Hídvégi, located in western Hungary (46°38′ N, 17°08′ E; 110 m a.s.l.), is an artificial lake system built in 1985 as a part of the Kis-Balaton Water Protection System, that has the overall function of retaining inorganic nutrients and total suspended solids carried by the Zala River into Lake Balaton [57]. Lake Hídvégi is a shallow, eutrophic to hypertrophic, predominantly open-water habitat (area: 18 km^2^, average depth: 1.1 m) partly covered by floating macrophytes, with *Trapa natans* L. as dominant species and some presence of *Nuphar lutea* (L.) Sm. and *Nymphaea alba* L. [46, 58]. Helophyte communities in the littoral zone are composed of reed beds (*Phragmites australis* (Cav.) Trin. ex Steud.) and cattail beds (*Typha* spp.).

Mantua lakes system, located in northern Italy plain (45° 10′ N, 10° 47′ E; 15 m a.s.l.), is composed of three dimictic shallow fluvial-lakes (area: 6.1 km^2^; average depth: 3.5 m), with two connected wetlands (upstream and downstream of lakes). The Superior, Middle and Inferior lakes are characterized by high turbidity and eutrophic conditions, and the water level is kept fixed to prevent flooding the city of Mantua. Macrophyte communities in the system are populated by both native (*T. natans, N. lutea*, *N. alba* in open water areas, and *P. australis* in wetlands) and alien species [46, 59, 60]: *Nelumbo nucifera* Gaertn., introduced into the lake around a century ago, and *Ludwigia hexapetala* (Hook. & Arn.) Zardini, H.Y. Gu & P.H. Raven, which started spreading here during the last decade.

Lake Varese, located in subalpine northern Italy (45° 49′ N, 8° 44′ E; 238 m a.s.l.), is a monomictic, eutrophic lake (area: 14.2 km^2^; average depth: 10.9 m) subject to high anthropic pressures and nutrient loads. The southern shores of the lake host extensive stands of floating macrophytes, mainly *T. natans* and *N. lutea*, with some presence of *N. alba* [61]. Some tracts of the littoral zone have been colonized in the last couple of decades by alien species, *N. nucifera* and *L. hexapetala*.

### Field measurements

Boat-based surveys were carried out in the three sites for 3 years (2016–2018), covering different times within the macrophyte growing season, spanning from late May to late July. Due to logistic and technical constraints, macrophyte beds sampled were not always the same ones in different years. During the surveys, leaf samples from six species (*L. hexapetala, N. nucifera, N. alba, N. lutea. P. australis, T. natans*) were measured and collected over various locations, to incorporate intra-site variability. Besides being among the most represented floating and emergent macrophytes in temperate Europe, the mentioned species show a clear dominance in the study sites, covering a vast majority (more than 90%) of the area occupied by aquatic and wetland vegetation. A summary of sampling locations for each species and date of survey is provided in Additional file 1: Table S1. Leaf samples, either floating or emergent above water, were collected from plants growing in dense (canopy fraction cover > 60%) and homogeneous stands, within 3 m of the water edge; from each plant sampled, the youngest, mature leaf, directly exposed to sunlight was taken for measurements.

#### Leaf spectral reflectance

Leaf reflectance in the visible to shortwave infrared range (350–2500 nm, with a spectral resolution of 3 nm for wavelengths under 1000 nm, and < 8 nm up to 2500) was measured using a portable full range spectroradiometer (SR-3500, Spectral Evolution, Lawrence, USA), following the protocol described in Tóth et al. [60]. After 20-min dark adaptation, leaves were laid on a flat neoprene plate (reflectance factor < 5%) to minimize background reflection of light transmitted through the leaves. Leaf reflected radiance was measured at contact using a probe equipped with a 5-W internal light source under near-steady state conditions, i.e. 60 s after removing the leaf clip. Each spectrum is the result of 10 averaged scans, and automatic integration time optimization was used, with a maximum allowed of 50 ms per scan. Leaf spectra were eventually calibrated to reflectance using reflected radiance from a Spectralon panel (Labsphere, North Sutton, USA; reflectance factor > 99% for wavelengths under 1500 nm, and > 95% up to 2500 nm) as reference.

#### Leaf photophysiology

Photophysiological traits of macrophytes were assessed using chlorophyll fluorescence measured with a PAM-2500 chlorophyll fluorometer (Heinz Walz GmbH, Germany) over the same leaves sampled for spectral reflectance measurements. Data were collected occasionally between 09:00 and 15:00, standard solar time. Relevant fluorescence yield data (initial fluorescence yield—F_0_, maximal fluorescence yield—F_m_) were measured following the protocol described in Tóth et al. [60] on mature, healthy-looking leaves after a dark-adapting period of 20 min with a pulse of a saturated light (630 nm, intensity 3000 μmol m^−2^ s^−1^). Photochemical PSII efficiency (F_v_/F_m_), coefficient of photochemical quenching (qP), coefficient of non-photochemical quenching (qN), maximum electron transport rate of the photosystem II (PSII) (ETR_max_), theoretical saturation light intensity (I_k_, and maximum quantum yield for whole chain electron transport (α were calculated using fluorescence yield data and a light response curve—with ETR measured as a function of photosynthetically active radiation (PAR; 11 steps between 5 and 787 μmol m^−2^ s^−1^)—described by an exponentially saturating equation [62]. Leaf absorbance was set at 0.84 for the calculation of ETR for all the species sampled.

#### Leaf biochemical and structural traits

Leaf pigments, as biochemical traits directly connected with photosynthetic apparatus, and leaf structural traits were measured on our samples to represent foliar economics expressing the inner trade-off between resource availability and structural investments, which lies at the core of the LES.

Two leaf discs (0.6 cm in diameter) were cut with a cork borer from each fresh leaf, in the vicinity of where chlorophyll fluorescence was measured. Disks were stored in aluminium foil at < 0 °C in a camping fridge until they were transferred to the laboratory, i.e. within maximum of 4 h.

Half of the discs sampled were stored at – 20 °C in a freezer, to be used for pigments extraction, following the protocol described in Tóth et al. [60]. Upon extraction, they were homogenised in liquid N_2_ in a grinder, subsequently extracted in acetone solution (80%), and stored in a fridge overnight. The extracts were centrifuged and the supernatant collected and stored at − 20 °C. The full spectra of absorbance of the extracts were measured between 400 and 750 nm using a spectrophotometer (Shimadzu UV-2401PC, dual-beam), at 1 nm resolution. Finally, pigment concentrations, i.e. chlorophyll-a (Chl-a), chlorophyll-b (Chl-b), and total carotenoids (Car) were calculated using empirical formulae [63] and reported on a leaf area basis (µg cm^−2^). Pigment ratios were then calculated, as Chl-a to Chl-b ratio (Ca/Cb) and total chlorophylls (Chl-a + Chl-b) to total carotenoids ratio (Chl/Car).

The other half of leaf discs were weighted with a precision balance (Mettler Toledo AB104, 0.0001 g accuracy) to measure their fresh weight, and were then dried in a ventilated oven at 70 °C for 48 h, after which the dry weight was measured using the same balance. From these data, dry matter content (DMC) and leaf mass per area (LMA) of each disc were calculated as the ratio of dry weight to fresh weight (g g^−1^), and the ratio of dry weight to disc area (g m^−2^) respectively.

#### Interspecific and intraspecific variability

Due to the non-normality of samples for some traits (and species) in our dataset, the variability of leaf traits across species was tested using non-parametric Kruskal–Wallis One Way ANOVA, and pairwise comparisons were performed using post-hoc Dunn’s test, with p-value adjustment computed according to Benjamini–Hochberg method. Intraspecific variability of selected traits was assessed by calculating the coefficient of variation (CV) of each trait for every species, using only leaf samples collected at peak of growth (mid-late July) to reduce differences due to phenology.

### Spectral indices and leaf traits

For each possible combination of two spectral reflectance bands measured as described in “Leaf spectral reflectance” section within the range 400–2500 nm (987 bands), the normalized difference spectral index (NDSI) was calculated using a custom-made R script [45]. NDSIs are frequently used in RS because they offer the advantage of summarizing spectra information, while reducing uncertainty due to sensor differences and atmospheric effects and bias due to differences in vegetation background [64–66], and are defined as:

1 $${NDSI}_{i,j}=\frac{{\rho }_{i}-{\rho }_{j}}{{\rho}_{i}+{\rho}_{j}}$$

where *ρ* is leaf reflectance measured at spectral bands *i* or *j*.

Using the same R script, the correlation (Pearson’s *r*) between each leaf parameter, i.e. photophysiology, pigments and structural trait, and every calculated NDSIs was calculated together with the corresponding p-value (p), accounting for multiple comparisons using p-adjustment based on Bonferroni method. Even if deviations from normality were observed for some traits in our dataset (see Additional file 1: Fig. S9), we opted for the use of squared Pearson’s *r* as a measure of effect in assessing the strength of NSDI-trait relations commonly found in related literature for the easy interpretability (starting from [67]), after some tests comparing this approach with alternative ones, i.e. traits transformation or non-parametric correlations, that showed its conservativeness in assessing the strength of NDSI-trait relations, while not changing substantially the selection of the optimal band combinations (Additional file 1: Figs. S2, S3). Correlation plots featuring the coefficient of determination (R_cal_^2^), calculated as the square of *r*, between each leaf parameter and the complete combinations of NDSIs derived from leaf reflectance were drawn for significant relations (p_adj_ < 0.01), in order to highlight optimal two-band combinations proxies of investigated leaf photophysiology (α, ETR_max_, I_k_, F_v_/F_m_, qN, qP), pigments pool (Chl-a, Chl-b, Car, Ca/Cb, Chl/Car), and structural traits (DMC, LMA).

### Hyperspectral modelling of leaf traits

Partial least-square regression (PLSR) modelling [68] was used to further explore the relations between leaf reflectance and selected leaf traits across floating and emergent macrophyte species, using package *pls 2.7,* implemented in R ([69, 70]. PLSR is a powerful tool for modelling vegetation parameters using spectral datasets because it is suitable for cases when the number of predictors is larger than that of observations and can handle multi-collinearity in predictor variables, as frequently happens with narrow-band hyperspectral data [55, 71].

In order to focus on meaningful relations, PLSR models were calibrated only for leaf traits that showed a minimum sensitivity to spectral reflectance, that is scoring a R_cal_^2^ against all combinations of NDSIs higher than 0.15 (p_adj_ < 0.01), while simultaneously excluding mutually correlated traits (i.e. scoring Pearson’s *r* vs. any other trait < − 0.5, or > 0.5). The number of PLSR components used for each calibrated model (with 30 components set as maximum, following preliminary tests) was optimized through minimization of the root mean square error of prediction (RMSEP) via leave-one-out cross-validation (LooCV). The best model for each trait was eventually selected by setting the number of PLSR components corresponding to minimum cross-validation RMSEP, and model performance was assessed comparing measured with PLSR predicted trait values through the coefficient of determination of measured vs. predicted traits via LooCV (R_CV_^2^) and the RMSE normalized based on the range of values for each trait (nRMSE). The relative importance of different wavelengths and spectral ranges to PLSR models calibrated for each variable was assessed by computing the Variable Importance of Projection (VIP; [42]).

### Airborne hyperspectral images

Airborne hyperspectral images of Lake Hídvégi and Mantua lakes system were acquired from the APEX imaging spectrometer [72] on 19 July 2014 (around 12:00 local time) and 27 September 2014 (around 13:45 local time), respectively. Visible to near-infrared range APEX data were processed, resulting in hyperspectral images composed of 98 spectral bands (425–905 nm), with 3–10 nm spectral resolution.

APEX data were calibrated to at-sensor radiance units and georeferenced based on the sensor’s GPS/IMU at 5 m spatial resolution on the ground. Surface reflectance was finally derived by applying atmospheric correction based on MODTRAN-4 code and optimized for water targets [73]. To diminish distortions due to canopy structure effects and the influence of mixture with canopy background in APEX-derived spectra, we used the vector normalization method developed by Feilhauer et al. [55] and we filtered results using previously derived spatialized information about LAI of macrophyte communities in the same sites [46], by excluding all pixels with LAI < 0.67 m^2^ m^−2^.

As a proof of concept for demonstrating the potential of hyperspectral remote sensing data in highlighting spatial patterns of macrophyte traits at fine scale, we have produced *bio-visualisation* maps as RGB composites of spectral proxies derived from APEX normalized surface reflectance bands, by calculating the NDSIs recognized as linked to selected traits from leaf spectroscopy-based analysis whose NDSI-trait relation scored an R_cal_^2^ > 0.4 within the spectral range covered by APEX.

## Results

### Variability of macrophyte leaf traits

**Fig. 1:**
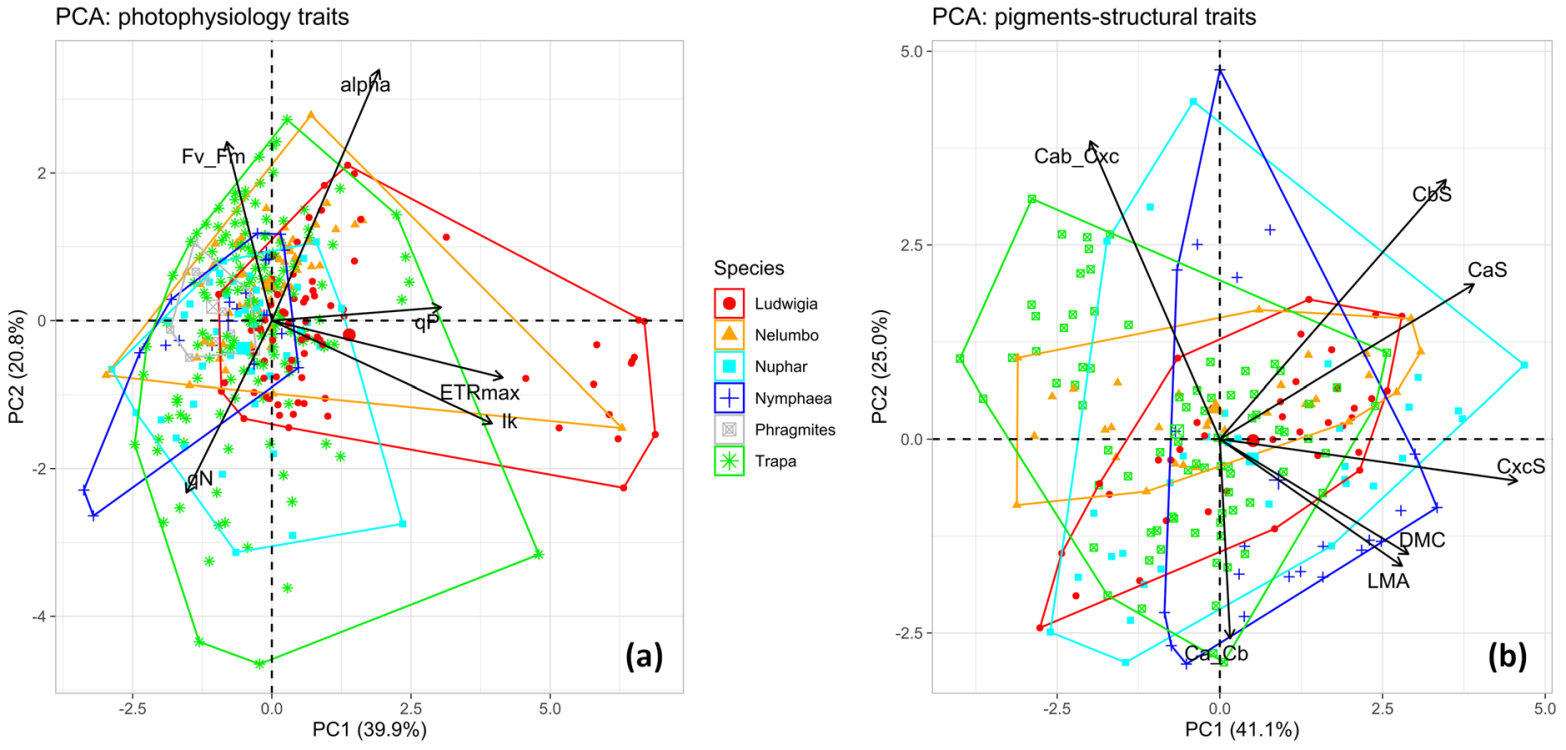
Bi-plots of the first two Principal Components of leaf photophysiological parameters (**a**) and foliar economics (**b**) expressed as pigments content and leaf structural traits, measured over all macrophyte species sampled. As visual guidance for data interpretation, minimum convex polygons grouping all points of a species are overlaid on both plots. alpha: maximum quantum yield for whole chain electron transport; ETRmax: maximum electron transport rate for PSII; Ik: theoretical saturation light intensity for PSII; Fv_Fm: PSII photochemical efficiency; qP: coefficient of photochemical quenching; qN: coefficient of non-photochemical quenching; CaS: chlorophyll a content; CbS: chlorophyll b content; Cxc: total carotenoids content; Ca_Cb: chlorophyll a to chlorophyll b ratio; Cab_Cxc: chlorophylls to carotenoids ratio; DMC: leaf dry matter content; LMA: leaf mass per area

Regarding traits related to photosynthetic performance, alien species showed significant differences with native ones, i.e. *N. nucifera* scored high α (adjusted p < 0.01 from all pairwise post-hoc Dunn’s tests; Additional file 1: Fig. S1), and *L. hexapetala* scored high ETR_max_, I_k_ and qP (adjusted p < 0.001 from all pairwise post-hoc Dunn’s tests; Additional file 1: Fig. S1). Moreover, regulated thermal dissipation of excess absorbed light (qN) was found to be slightly lower in *N. nucifera* and *L. hexapetala*, as well as in *T. natans*, compared to nymphaeids (*N. lutea* and *N. alba*) and *P. australis* (Fig. 1; Additional file 1: Fig. S1). *P. australis* showed quantum efficiency of photosystem II (F_v_/F_m_) slightly higher than all other species, except *N. alba* (p < 0.05 from pairwise post-hoc Dunn’s tests; Additional file 1: Fig. S1).

*Ludwigia hexapetala* displayed Chl-a values slightly higher than native species (p < 0.05 from all pairwise post-hoc Dunn’s tests; Additional file 1: Fig. S1), while no significant difference across species was observed for Chl-b, and the lowest carotenoid content was found in *N. nucifera* and *T. natans* (p < 0.05 from all pairwise post-hoc Dunn’s tests; Additional file 1: Fig. S1). Nymphaeids got the lowest Ca/Cb ratio among species (p < 0.05 from pairwise post-hoc Dunn’s tests), with *L. hexapetala* and *T. natans* occupying the high band of scores (Additional file 1: Fig. S1). A strong level of segmentation at species level was shown for Chl/Car ratio (p < 0.001, Kruskal–Wallis One Way ANOVA): *N. nucifera* and *T. natans* exhibited the highest scores (p < 0.001 from pairwise post-hoc Dunn’s tests) and *L. hexapetala*, *N. lutea*, *N. alba* followed in decreasing Chl/Car (Additional file 1: Fig. S1).

Compared to native species, leaf DMC was higher (p < 0.001 from all pairwise post-hoc Dunn’s tests) and LMA lower (p < 0.001 from all pairwise post-hoc Dunn’s tests) for both *L. hexapetala* and *N. nucifera* (Fig. 1; Additional file 1: Fig. S1). Inter-species DMC patterns tend to be similar to those of Chl/Car, with the exception of *T. natans*, and strong differentiation in LMA was observed among species, with nymphaeids scoring the highest values and *T. natans* spanning the widest range (Fig. 1; Additional file 1: Fig. S1).

Leaf data collected at peak of growth (i.e. samples collected in July) exhibited a notable degree of intraspecific variability for most of the traits (Fig. 1), as maximum scores of CV across all species was larger than 0.29, with the only exceptions of α and F_v_/F_m_ (Table 1). *P. australis* presented the lowest variable set of photophysiology traits except for qP (CV < 0.19), while nymphaeids had the most variable traits (α, qP, Ca/Cb and Chl/Car for *N. alba*; Chl-a, Chl-b and Car for *N. lutea*). *L. hexapetala* showed very high plasticity in photophysiology parameters ETR_max_, I_k_, and qN (CV > 0.32), and *T. natans* scored highest intraspecific ranges of DMC and LMA (CV > 0.29).

**Table 1:** Coefficient of variation (CV) of all leaf traits measured over macrophytes species sampled (highest scores across species for each trait are in bold, lowest in italic) at peak of growth conditions (July)

*Species*	Photophysiology parameters						Leaf pigments					Leaf structure	
	α	ETR_max_	I_k_	F_v_/F_m_	qN	qP	Chl-a	Chl-b	Car	Ca/Cb	Chl/Car	DMC	LMA
*L. hexapetala*	0.134	**0.886**	**0.695**	0.060	**0.325**	0.111	0.303	0.295	*0.243*	0.098	0.123	0.160	*0.163*
*N. nucifera*	0.139	0.277	0.205	0.046	0.263	0.194	0.286	0.266	0.299	*0.089*	*0.093*	0.203	0.216
*N. lutea*	0.149	0.279	0.255	0.064	0.133	0.242	**0.313**	**0.434**	**0.307**	0.186	0.302	*0.086*	0.171
*N. alba*	**0.202**	0.396	0.244	*0.029*	0.183	**0.378**	*0.190*	0.368	0.293	**0.293**	**0.363**	0.136	0.217
*P. australis*	*0.074*	*0.172*	*0.187*	0.044	*0.072*	0.156							
*T. natans*	0.150	0.318	0.389	**0.099**	0.302	0.214	0.196	*0.185*	0.258	0.098	0.162	**0.292**	**0.433**

### Spectral indices as proxies for macrophyte leaf traits

**Fig. 2:**
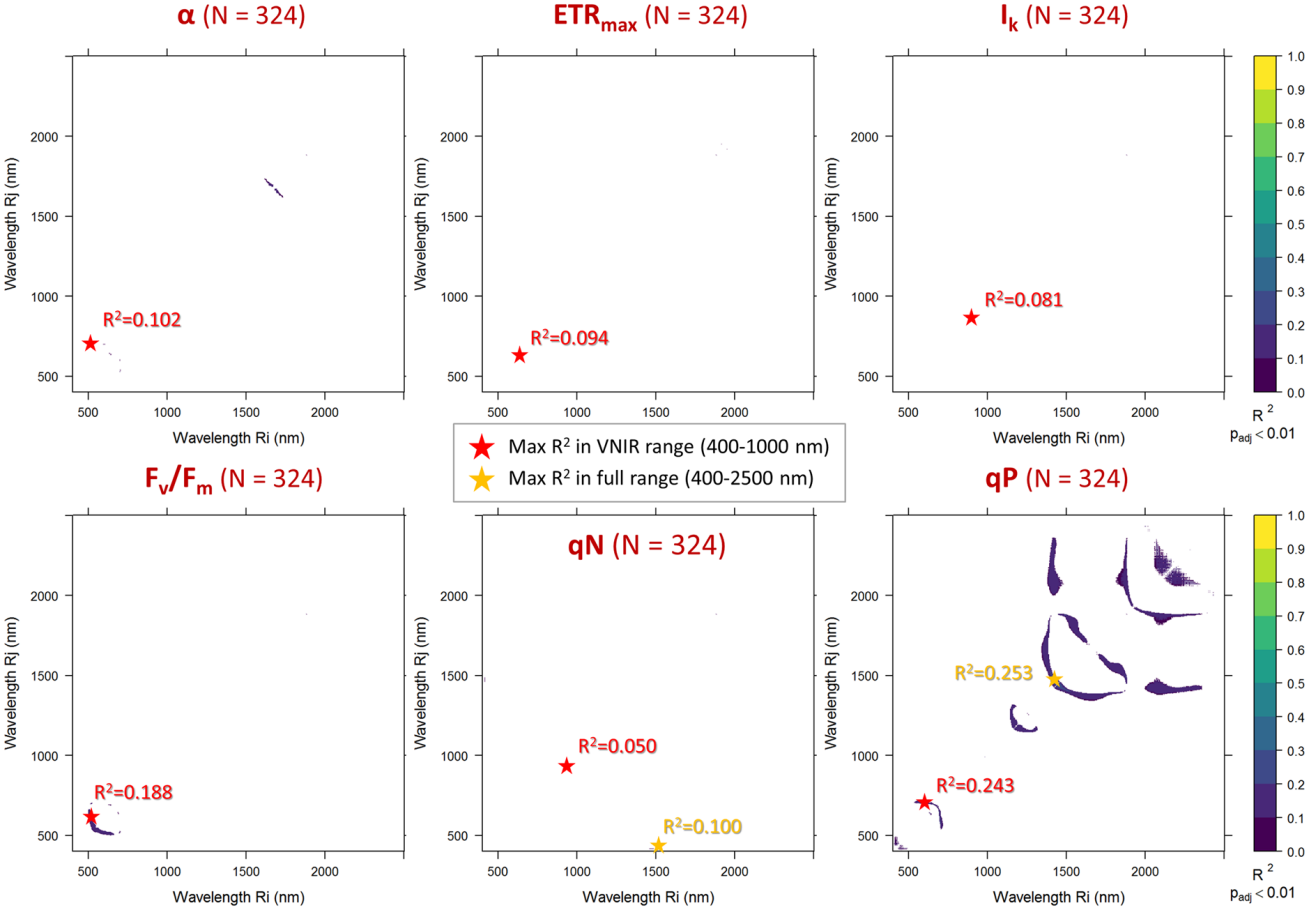
Statistically significant (p < 0.01, Bonferroni adjusted) NDSI correlations with leaf photophysiology parameters measured on all macrophyte species sampled (N = 324), over the full spectral range covered (400–2500 nm)

**Fig. 3:**
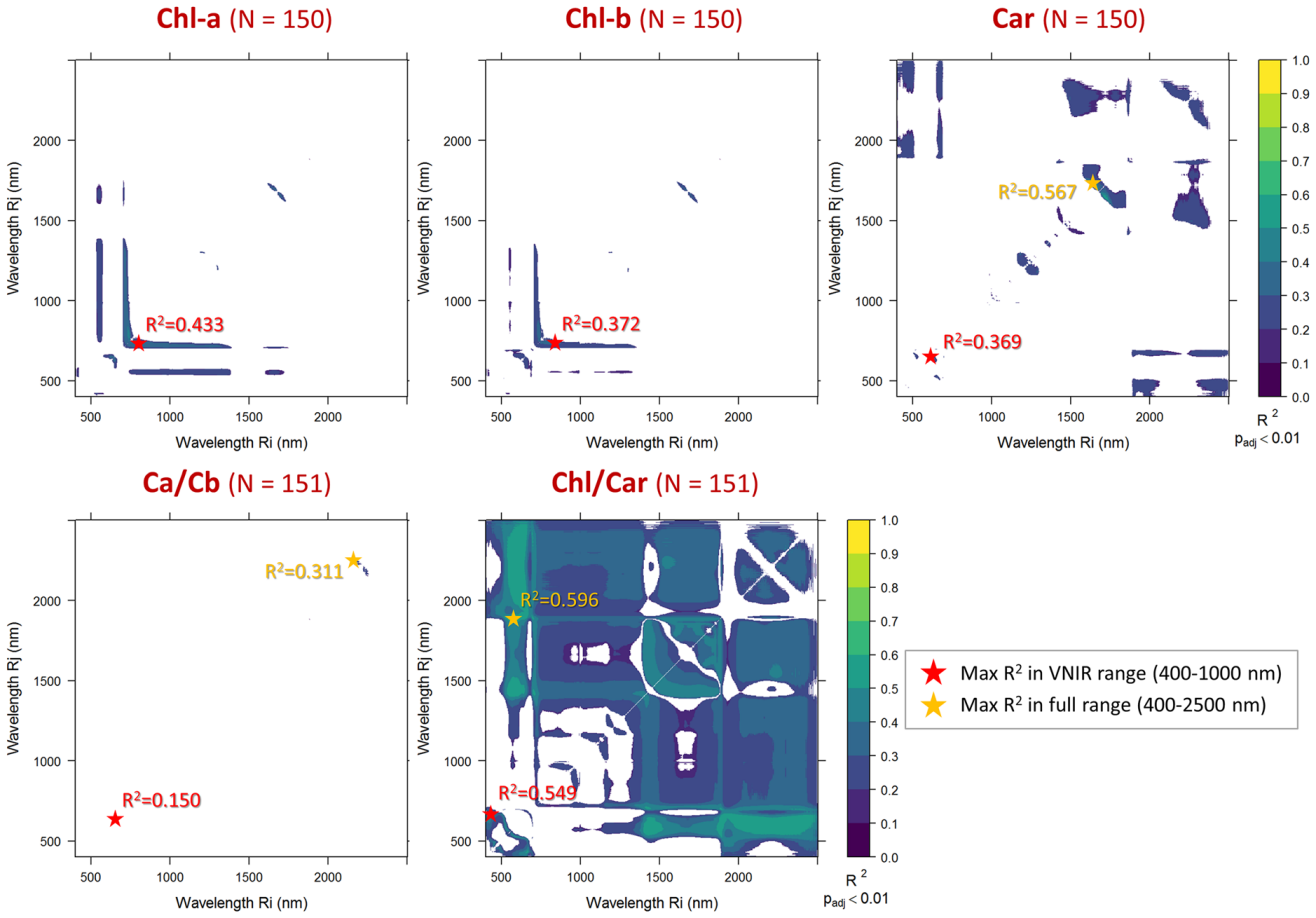
Statistically significant (p < 0.01, Bonferroni adjusted) NDSI correlations with leaf pigments content (on leaf area basis, N = 150) and their balance (N = 151) measured on all macrophyte species sampled, over the full spectral range covered (400–2500 nm)

**Fig. 4:**
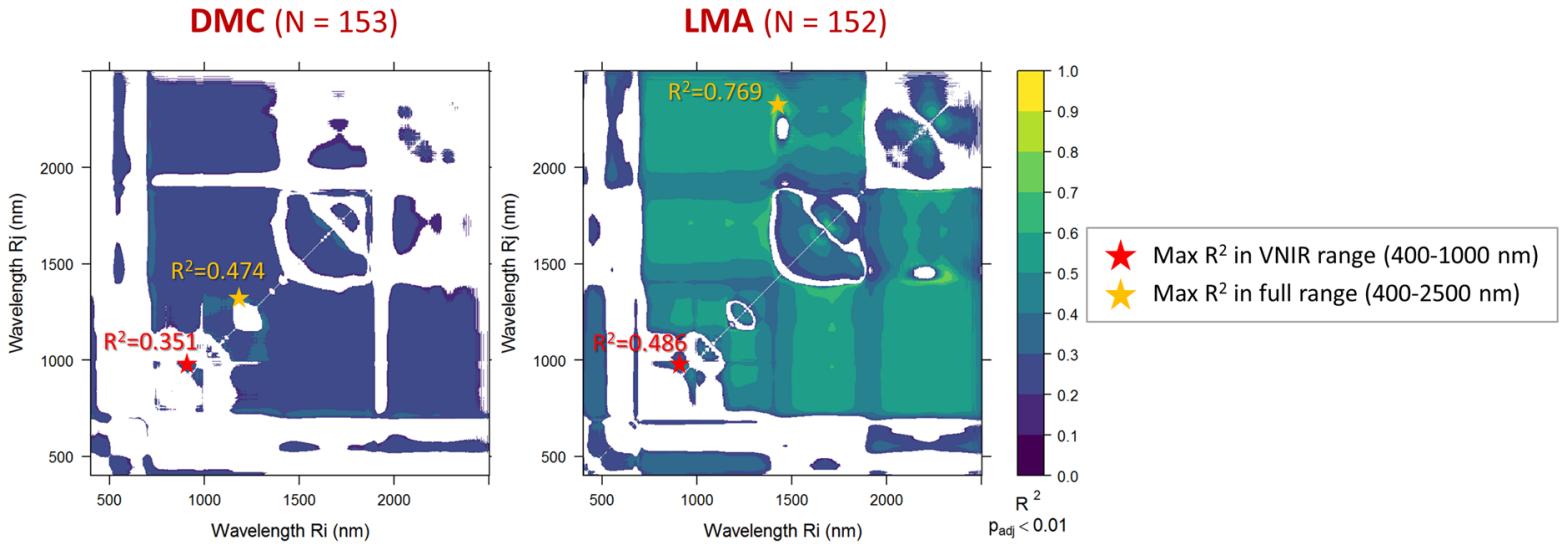
Statistically significant (p < 0.01, Bonferroni adjusted) NDSI correlations with leaf structure straits measured on all macrophyte species sampled, over the full spectral range covered (400–2500 nm): leaf dry matter content (DMC, N = 153) and leaf mass per area (LMA, N = 152)

The correlations of every two-band combination NDSI correlations with investigated leaf traits measured on all macrophyte species sampled are highlighted in Figs. 2, 3, 4 showing the optimal spectral proxies for each trait in the visible to near-infrared (VNIR) spectral range (400–1000 nm) and in full spectral range (400–2500 nm), i.e. extending to shortwave infrared (SWIR) wavelengths.

The heterogeneity of measured samples not only in terms of species but also in terms of location (three study sites) and time of sampling (3 years, different months)—which implies that our dataset includes a high variability in genetic features, seasonal cycles, stage of growths, plant conditions and environmental settings (N = 324)—overall resulted in relatively low correlation patterns (R_cal_^2^ < 0.26) between leaf reflectance derived NDSI and measured photophysiological traits (Fig. 2). In particular, across all species, negligible correlations with any NDSI combinations were shown for α and qN (overall maximum R_cal_^2^ = 0.10, even if p_adj_ < 0.01), while results were not statistically significant (p_adj_ ≥ 0.01) for ETR_max_ and I_k_. Slightly higher correlations, but still moderately weak, are shown for F_v_/F_m_ and qP, with R_cal_^2^ peaking at 0.19 and 0.25 (p_adj_ < 0.01), respectively (Fig. 2). The optimal NDSI for F_v_/F_m_ combined reflectance in the range of green visible light (524 nm, 581 nm), while for qP the best spectral combinations lay in the SWIR range, roughly around 1400–1500 nm.

Notwithstanding the abovementioned heterogeneity that biases the reflectance-photophysiology relations, correlations between pigments and NDSI computed from leaf reflectance across species are tangible, as peak R_cal_^2^ found was always higher than 0.3 (Fig. 3, p_adj_ < 0.01). In particular, NDSIs and Chl-a were found to be mutually correlated, particularly in the red-edge range (maximum R_cal_^2^ = 0.43 for NDSI_775,740_, N = 150). As Chl-b is highly correlated with Chl-a in our dataset (*r* = + 0.80), optimal band combinations are found around the same range highlighted for Chl-a (795 nm, 740 nm), yet with slightly weaker correlation (maximum R_cal_^2^ = 0.37, N = 150). Among pigments, Car (N = 150) showed the strongest correlations, peaking for NDSIs featuring band combinations in the SWIR range (maximum R_cal_^2^ = 0.57 for NDSI_1644,1720_), while correlations decreased if the spectral range was limited to VNIR (maximum R_cal_^2^ = 0.37). Ca/Cb ratio (N = 151) showed the weakest correlations of all pigment traits, with a peak R_cal_^2^ = 0.31 in the SWIR range (for NDSI_2181,2241_). Chl/Car ratio (N = 151) is instead very well surrogated by NDSIs combining visible to SWIR reflectance (maximum R_cal_^2^ = 0.60 for NDSI_611,1892_); slightly lower correlations, yet still the highest among all pigments traits measured (maximum R_cal_^2^ = 0.55), were scored restricting the spectral range to VNIR range, where optimal NDSI for Chl/Car combined reflectance in 433 nm and 665 nm, which indeed roughly correspond to absorption peaks of chlorophyll-a [66].

Relatively stronger correlations were scored between NDSIs derived and leaf structural traits measured, connected to leaf economics, DMC and LMA (Fig. 4). DMC (N = 153) demonstrated a good sensitivity to leaf reflectance in the NIR to SWIR ranges (p_adj_ < 0.01), with R_cal_^2^ up to 0.47 (for NDSI_1196,1308_), and slightly weaker scores in the shorter VNIR wavelengths (maximum R_cal_^2^ = 0.35 for NDSI_929,941_). Among all leaf traits, LMA (N = 152) scored the highest correlations with NDISs, with a large subset of band combinations showing R_cal_^2^ > 0.6 in the NIR to SWIR ranges, and a peak R_cal_^2^ = 0.77 (p_adj_ < 0.01) when two bands centred at 1415 and 2305 nm were used (Fig. 4).

### Leaf reflectance-traits relations

After having assessed mutual correlations of measured leaf traits, excluding the ones with |*r*|> 0.5 (Additional file 1: Fig. S9), PLSR models were calibrated for traits scoring non-negligible correlations with best performing NDSIs presented in the previous section (R_cal_^2^ > 0.15): F_v_/F_m_, qP, Chl-a, Ca/Cb, Chl/Car, DMC, LMA.

**Fig. 5:**
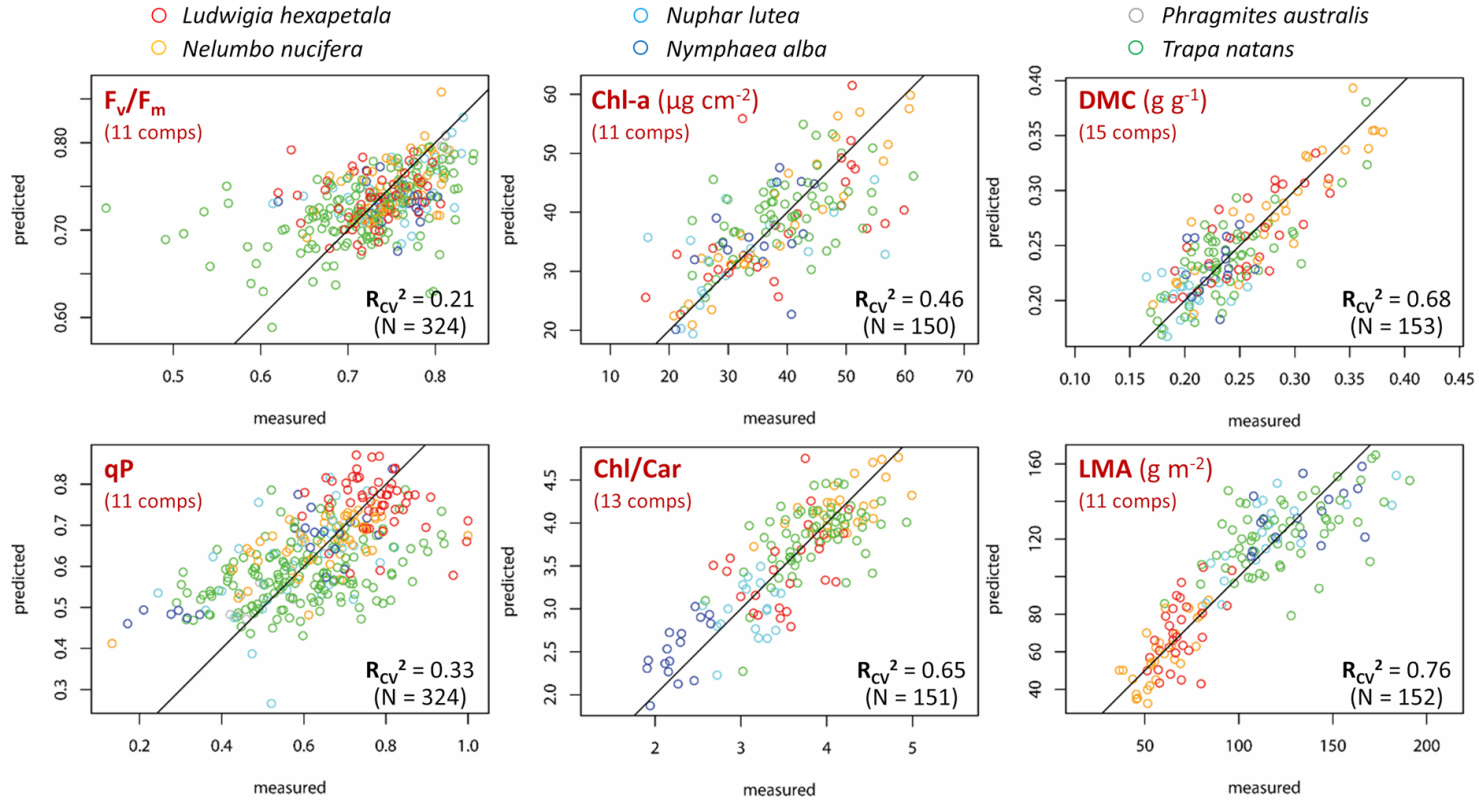
Comparison of leaf traits measured and predicted by best fit PLSR models of selected traits for all macrophyte species sampled (N = 150–324), computed via leave-one-out cross-validation

The best fit PLSR models for F_v_/F_m_ and qP (N = 324) require 11 components, with RMSEP of 0.053 and 0.125 respectively (Additional file 1: Fig. S10). Matching between measured and predicted photophysiology traits was quite low for F_v_/F_m_ (R_CV_^2^ = 0.21) and qP (R_CV_^2^ = 0.33), even if estimation error seems to be reducing when extreme values—probably due to stress conditions non visibly detected when leaves were chosen for sampling—are excluded, i.e. for F_v_/F_m_ > 0.65, and 0.4 < qP < 0.8 (Fig. 5).

**Fig. 6:**
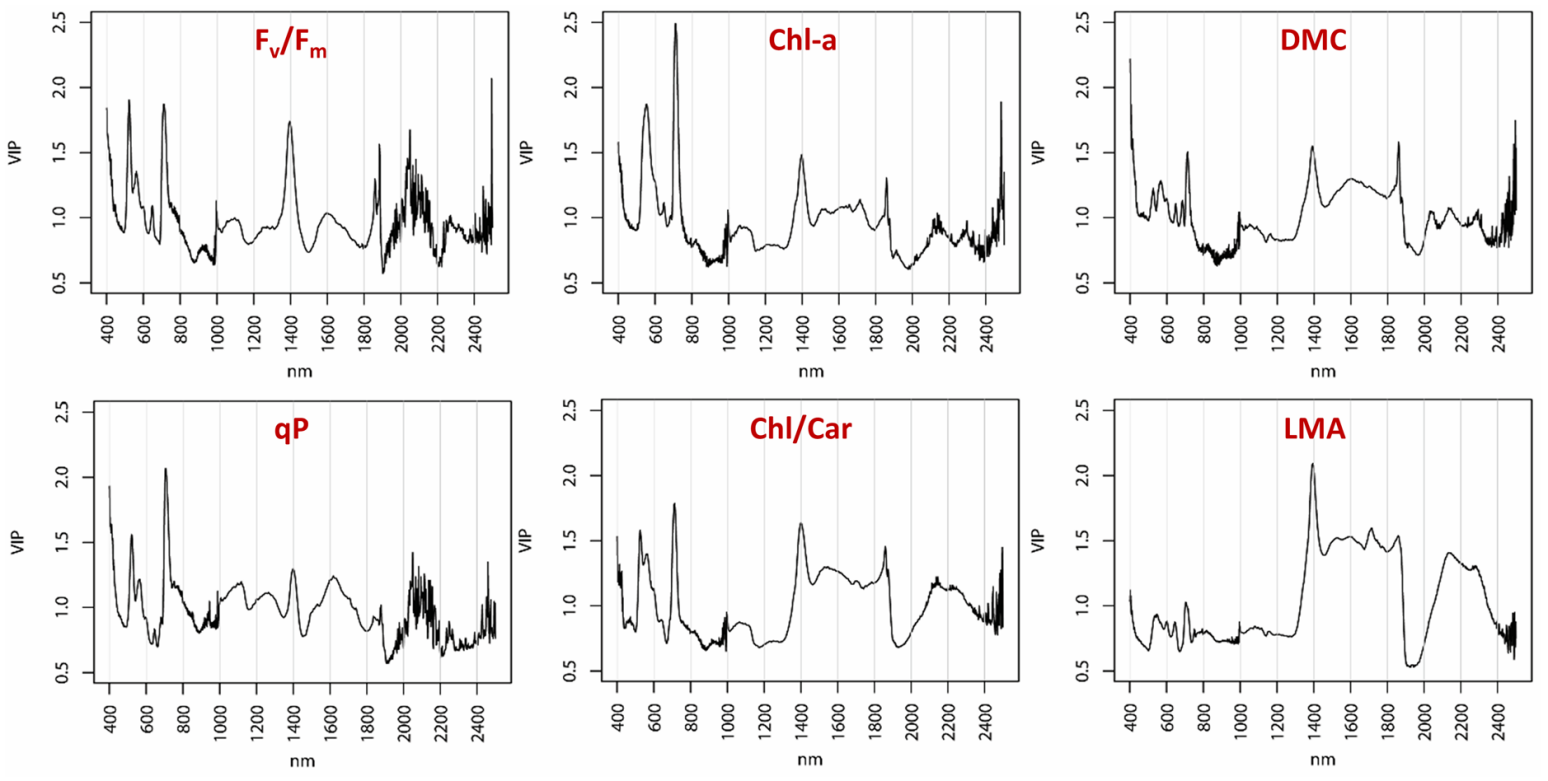
Variable Importance of Projection (VIP) scores of PLSR models of selected leaf traits for all macrophyte species sampled, computed for each wavelength, over the full spectral range covered (400–2500 nm)

PLSR models based on leaf reflectance are quite effective in predicting macrophyte Chl-a content (N = 150) and Chl/Car ratio (N = 151) across species, with best fit models requiring 11 and 13 components (Fig. 5, Additional file 1: Fig. S10) respectively, achieving RMSEP of 7.52 µg cm^−2^ (R_CV_^2^ = 0.46, nRMSE = 16.6%) and 0.393 (R_CV_^2^ = 0.65, nRMSE = 12.7%). Figure 6 shows that VIP scores are high around 550–560 nm and 705–710 nm for Chl-a model and around 705–710 nm and 1400 nm for Chl/Car model. Conversely, modelling performance for Ca/Cb is the lowest among traits considered here (R_CV_^2^ = 0.20), suggesting that spectral reflectance might not be a good proxy for this trait, at least for macrophyte species.

DMC (N = 153) and LMA (N = 152) are the leaf traits better surrogated by reflectance-based models (Fig. 5, Additional file 1: Fig. S10), with matching between measured and PLSR predicted scores up to RMSEP = 0.026 g g^−1^ (nRMSE = 12.1%) for DMC (R_CV_^2^ = 0.68, with 15 components) and up to RMSEP = 18.6 g m^−2^ (nRMSE = 11.7%) for LMA (R_CV_^2^ = 0.76, with 11 components), across the whole range of measures in our dataset. VIP scores for DMC model are high around 400–410 nm and 1390–1400 nm, while for LMA model the wavelengths in VNIR range do not contribute much to prediction and peak VIP scores are found between 1400 and 1850 nm, as well as between 2120 and 2300 nm (Fig. 6).

### Mapping spectral functional proxies from hyperspectral images

The potential for application of the main findings described in previous sections was tested by producing spatial variability maps for selected spectral proxies of macrophyte traits deriving specific NDSIs from airborne hyperspectral data (APEX) collected over Lake Hídvégi and Mantua lakes system during different times of summer in 2014. Maps are derived only for those spectral indices showing best matching with in situ measured data in the spectral range of APEX data (425–905 nm): i.e. NDSI_775,740_ as a proxy of Chl-a content (R_cal_^2^ = 0.43), NDSI_433,665_ as a proxy of Chl/Car ratio (R_cal_^2^ = 0.55), and NDSI_690,500_ as a proxy of LMA (R_cal_^2^ = 0.44).

**Fig. 7:**
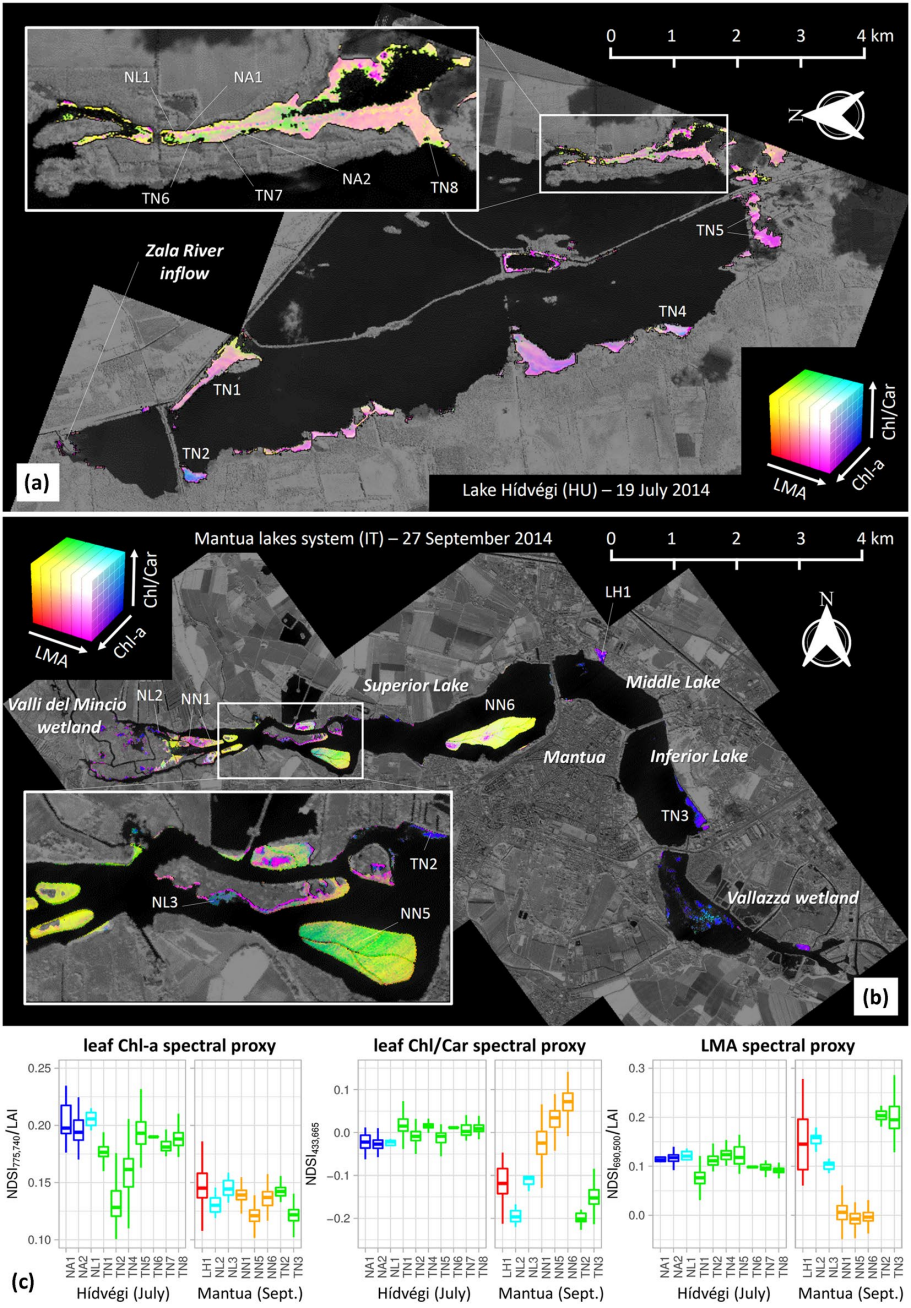
Maps of spectral proxies (linked to selected traits from leaf spectroscopy) derived from airborne hyperspectral (APEX) data, showing RGB combinations of best two-band combined indices (NDSI) in the range covered by APEX data for Chl-a content (NDSI_740,775_), Chl/Car ratio (NDSI_433,665_), and LMA (NDSI_690,500_): **a** macrophyte stands in Lake Hídvégi; **b** macrophyte stands in Mantua lakes system; **c** box plots of spectral proxies for individual mono-specific stands in both sites (indicated in panels **a**, **b**). LH: *Ludwigia hexapetala*; NN: *Nelumbo nucifera*; NL: *Nuphar lutea*; NA: *Nymphaea alba*; TA: *Trapa natans*

The application of NDSI proxies using RS data inevitably bring to more or less significant biases, reflecting into the reliability of plant functional traits retrieved, which are due to complex combinations of factors, including: vegetation fractional cover and mixture with canopy background, density and structure effects (e.g. leaf orientation), reflectance anisotropy, and atmospheric effects [32]. All the more so, spectral data measured from APEX airborne imager, with pixel size in this case of 5 m, are inherently measuring the response of macrophyte beds at canopy scale, and application of proxies derived and assessed at leaf scale to some extent hampers the absolute matching of observation scales for spectral reflectance and plant traits. In deriving the maps of selected spectral proxies in Lake Hídvégi and Mantua lakes system (Fig. 7), we have partly tackled these distortion factors using reflectance normalization and masking our pixels with low canopy density (LAI < 0.67 m^2^ m^−2^). As a proof of concept of the usefulness of our findings to investigate spatial patterns of macrophyte functional diversity, maps shown in Fig. 7 are clearly capturing visible patterns of relative variability in macrophyte communities at within-system scale that can inform about specific features of macrophytes growing in Lake Hídvégi and Mantua lakes system.

## Discussion

Due to their sedentary nature, plant survival depends on the possibility to acclimate or adapt to the given environmental conditions and biogeochemical processes of the area. The persistence of a species or a population is shaped by complex interactions of ecological and evolutionary attributes, and their changes during the growing season [74, 75]. Within the large variability typical of their habitat, macrophytes can establish and persist either by tolerating substantial environmental changes through their phenotypic plasticity or by shifting community composition when approaching a peripheral situation [76]. Macrophyte leaf trait data collected—covering different sites (three shallow lakes and wetlands in Europe), times (late May to late July), and seasons (3 years)— showed a high degree of heterogeneity, both within and among species sampled.

Some of the studied traits have evolutionary relevance: our data show that species with remarkable photochemical properties—*N. nucifera* with a higher maximum quantum yield of photosynthetic electron transport (α), *L. hexapetala* with the highest maximum electron transport rate, saturation irradiance and photochemical quenching, and *T. natans* with the highest range of variability in quantum yield of PSII—correspond to those with a prominent presence in terms of stands area and spread in the study sites. Leaf pigments content and leaf structural traits rather showed ecological relevance, as truly floating-leaved species (*N. lutea* and *N. alba*) had lower Chl/Car ratio to make better use of underwater diffused light, while species with some leaves emerging above the water (*L. hexapetala*, *N. nucifera*) showed higher dry matter content and lower LMA.

Alien, invasive species tend to be photochemically more efficient (higher α, ETR_max_, or I_k_) and to have lower LMA compared to native species growing in the same sites, which is in accordance with published findings on terrestrial plants [77, 78]. Differently from what was found on a mass basis in previous works [60], foliar pigment content on an area basis did not separate alien and native plants, implying that interspecific differentiation in studied macrophytes is driven more by pigment proportions, and therefore by the ability of species to adapt to a given environment and maximise both light absorption and photoprotection [79, 80]. These show the wide spectrum of macrophyte functional plasticity, but also hints at the individualistic responses of macrophytes to physical, chemical and anthropogenic characteristics of their environment.

Remote sensing spectroscopy is nearly the perfect tool to quantitatively assess the variability and specific trait distributions over large areas both within monospecific stands or mixed communities [46, 81], thus promoting functional studies focusing on inland water systems and connected ecotones and adding to literature that is considerably biased toward terrestrial ecosystems [82]. For this to work, a robust link between selected traits and spectral reflectance needs to be established starting from the fundamental leaf scale. Our results show that a suite of leaf functional traits, connected with light absorption and scattering mechanisms and representative of foliar economics trade-off, can be effectively modelled across floating and emergent macrophyte species based on leaf reflectance, thus complementing works performed in terrestrial ecosystems [32, 35, 39, 83], even under moderate to strong environmental heterogeneity, while photophysiology traits based on chlorophyll fluorescence measures could not be surrogated by leaf spectra with the same performance.

Best results were obtained via reflectance-based PLSR models for structural traits (leaf DMC and LMA), which constitute synthetic descriptors of leaf morphology, in particular the inner structure, mediating the effects of scattering mechanisms with water absorption [84]. These patterns generally confirm what has been documented from previous works on terrestrial plants at macro spectral ranges, i.e. in the SWIR [51, 52, 85–87], but with specific differences in optimal wavelengths to be used, possibly because of leaf structural peculiarities of aquatic plants [47]. Notably, in aquatic plants the accuracy of spectrally modelled LMA does not seem to depend on the magnitude of water content, deviating from the results of Riaño et al. [84], based on terrestrial species.

Complementing leaf structural traits in describing the trade-off at the core of the LES concept, leaf reflectance proved to be a good predictor of biochemistry-related traits in macrophytes, represented here by pigments pools and ratios. Chl-a content and Chl/Car ratio were modelled with good reliability based on leaf spectroscopy and PLSR. Pigments are more directly related to spectral reflectance because of their primary function of interacting with light and their location within the leaf structure—i.e. in the first, adaxial strata aquatic plant leaves, as shown by Borsuk and Brodersen [88] for chlorophylls in the case of *Eichornia crassipes* (Mart.) Solms. Optimal wavelengths for Chl-a predictions were found in the red-edge range (i.e. 700–800 nm), similar to what extensively documented for terrestrial plants [52, 85, 87, 89], with VIP of PLSR model peaking around 705–710 nm. Best performing spectral combinations for Ca/Cb ratio found around 2200 nm is possibly driven by the link between Ca/Cb and N content [90], which we did not measure and it is known to show absorption features around 2180 nm from previous works [91, 92].

Contrary to what was highlighted for leaf economics traits, reflectance-based PLSR models for photophysiological parameters were found to be under-performing across the species sampled. Best results were scored for F_v_/F_m_ (R_CV_^2^ = 0.21), with optimal spectral range very similar to photochemical reflectance index (PRI) [93], and qP (R_CV_^2^ = 0.33) roughly around 1400–1500 nm. These figures contrast some recent findings of site-specific studies in crops and tree species [56, 94, 95], and suggest specificities in aquatic plants, possibly connected to high heterogeneity in studied macrophytes, that hampers the generality of photophysiology-reflectance link across species (Additional file 1: Figs. S4–S8). Our results demonstrate that spectral reflectance, relatively straightforward to measure quantity at ecosystem scale even in aquatic environments, can be used via normalized indices or PLSR modelling for reliably estimating specific macrophyte leaf traits (Chl-a, Chl/Car, DMC and LMA), strongly connected to variability expressed within the LES in terms of trade-offs between structural investment and photosynthetic efficiency [96, 97]. This complements previous works based on terrestrial plants (e.g. [32, 38–42]) through new empirical data spanning over different species, seasonal, environmental conditions (sites) and phenological stages.

These findings provide the basis for using leaf spectra as a surrogate for high-throughput assessment of variability in macrophyte traits over scales and gradients and support the extension of the reflectance-based trait modelling to RS spectroscopic data for enhancing the level of detail of functional diversity analysis. In particular, the possibility of using spectral proxies for modelling macrophyte LMA opens intriguing perspectives for future research in aquatic species diversity and functioning and ecological applications of RS, aiming at investigating spatial and temporal gradients, helping to fill some of the gaps recently identified by Pan et al. [98]. The approach could be even extended to plant science in general, when paired with works already performed on terrestrial species [41]. Integrating high-resolution leaf reflectance spectroscopy with techniques based on Raman spectroscopy could potentially be highly profitable for further enhancing the study of processes and functioning of vegetation, including macrophytes [99].

Spatial-wise information derived from airborne hyperspectral data for spectral proxies connected to macrophyte traits can provide intuitive, realistic and detailed bio-visualisation of vegetation diversity and connected processes. Spatial patterns of variability in spectral proxies highlighted in Fig. 7 show differences at the site scale, due to both inter- and intra-species differentiation (e.g. *T. natans* in Lake Inferior, *N. nucifera* in Lake Superior within Mantua lakes system, Fig. 7b), as well as considerable variability at the community scale, indicating intra-specific trait plasticity (e.g. NDSI_775,740_ and NDSI_690,500_ of *T. natans* in Lake Hídvégi, as well as NDSI_433,665_ for *N. nucifera* in Mantua lakes system, Fig. 7c). Part of these differences are not only due to species composition but also to seasonal differences: APEX data collected over Lake Hídvégi in the middle of July represent macrophyte conditions at peak of growth, similar to most of the data we sampled in situ, while data for Mantua lakes system, acquired in late September, provide a static image of macrophyte communities in early to late senescence phase, depending on species, hence the high heterogeneity in NDSI_433,665_, linked to Chl/Car ratio (Fig. 7c).

Indeed, the heterogeneity highlighted for macrophyte communities of Mantua lakes system and Lake Hídvégi could be hardly seen with punctual measurements of macrophyte traits, and yet it is captured in a systematic, synoptic and quantitative way by testing leaf reflectance-traits relations we found on remotely sensed imaging spectroscopy data, informing us on spatial and temporal functional variability. Although some interesting case studies in this respect have been recently documented (e.g. [81, 100, 101]), they were limited to one or few systems and few aquatic vegetation characteristics (functional types, phenology metrics) and wider applications are needed.

## Supplementary Information

**Additional file 1: Table S1.** Summary of in situ samples collected for this study. **Figure S1.** Violin plots (with encompassed box plots) showing range and distribution of all leaf traits—photophysiological parameters, biochemistry (pigments) and leaf structure traits—measured over 6 macrophyte species (LH = *Ludwigia hexapetala*; NN = *Nelumbo nucifera*; NL = *Nuphar lutea*; NA = *Nymphaea alba*; PA = *Phragmites australis*; TN = *Trapa natans*). Plots show significant differences (p < 0.05) in pairwise comparison performed via Dunn’s post-hoc test with Benjamini–Hochberg adjustment. **Figure S2.** Comparison of NDSI correlations (p < 0.01, Bonferroni adjusted) with ETR_max_ for all samples (N = 324), in the visible to near-infrared spectral range (400–1000 nm): computed using Pearson’s *r* and Spearman’s *ρ* on raw data (top row), or Pearson’s *r* based on raw and transformed ($1/\sqrt{{ETR}_{max}}$) data (bottom row). **Figure S3.** Comparison of NDSI correlations (p < 0.01, Bonferroni adjusted) with LMA for all samples (N = 152), in the full spectral range (400–2500 nm): computed using Pearson’s *r* and Spearman’s *ρ* on raw data (top row), or Pearson’s *r* based on raw and transformed ($\sqrt{LMA}$) data (bottom row). **Figure S4.** Statistically significant (p < 0.01, Bonferroni adjusted) NDSI correlations with leaf photophysiology parameters measured on all macrophyte species sampled (N = 324) in the visible to near-infrared spectral range (400–1000 nm). **Figure S5.** Statistically significant (p < 0.01, Bonferroni adjusted) NDSI correlations with leaf photophysiology parameters measured on *Ludwigia hexapetala* samples (N = 53) in the visible to near-infrared spectral range (400–1000 nm). **Figure S6.** Statistically significant (p < 0.01, Bonferroni adjusted) NDSI correlations with leaf photophysiology parameters measured on *Nelumbo nucifera* samples (N = 55) in the visible to near-infrared spectral range (400–1000 nm). **Figure S7.** Statistically significant (p < 0.01, Bonferroni adjusted) NDSI correlations with leaf photophysiology parameters measured on *Nuphar lutea* and *Nymphaea alba* samples (N = 57) in the visible to near-infrared spectral range (400–1000 nm). **Figure S8.** Statistically significant (p < 0.01, Bonferroni adjusted) NDSI correlations with leaf photophysiology parameters measured on *Trapa natans* samples (N = 154) in the visible to near-infrared spectral range (400–1000 nm). **Figure S9.** Correlation matrix of leaf traits measured over 6 species. Pairwise scatter plots are shown in the lower left half, histograms are shown on the diagonal, and the coefficient of correlation (Pearson’s r) of each pair of traits is shown in the upper right half, including info about its significance level (*p < 0.05, **p < 0.01, ***p < 0.001). **Figure S10.** Variation of root mean square error of prediction (RMSEP) with PLSR model components, computed against the full dataset (training or leave-one-out cross-validation) for selected leaf traits estimated from leaf spectral reflectance for all macrophyte species sampled. **Figure S11.** Comparison of distribution of selected spectral proxies (NDSI) derived from APEX data at Lake Hídvégi and Mantua lakes system for mono-specific stands and connected leaf traits measured in our dataset (at peak of growth conditions). Data points represent median scores and whiskers delimit the extremes of values observed. Linear regression lines and their coefficient of determination (R^2^) separating stands in senescence phase at the time of overflight over Mantua site (27 September 2014) are superimposed on the graphs. LH_yel: *Ludwigia hexapetala* (with signs of chlorotic conditions); NN_sen: *Nelumbo nucifera* (in early senescence phase); NL: *Nuphar lutea*; NA: *Nymphaea alba*; TN: *Trapa natans*; TN_sen: *Trapa natans* (around senescence conditions).

## Data Availability

The datasets generated and/or analysed during the current study will be made available in public repositories upon publication: leaf traits data will be deposited in Dryad (datadryad.org); leaf reflectance spectra will be deposited also in EcoSIS (ecosis.org).
