# Supplementary material for: Leaf reflectance can surrogate foliar economics better than physiological traits across macrophyte species
Source: Plant Methods. 2021 Nov 10;17:115. doi: 10.1186/s13007-021-00816-4 (PMC8582205; doi:10.1186/s13007-021-00816-4)
Supplement: Supplementary file 1 — Additional file 1: Table S1. Summary of in situ samples collected for this study. Figure S1. Violin plots (with encompassed box plots) showing range and distribution of all leaf traits—photophysiological parameters, biochemistry (pigments) and leaf structure traits—measured over 6 macrophyte species (LH = Ludwigia hexapetala; NN = Nelumbo nucifera; NL = Nuphar lutea; NA = Nymphaea alba; PA = Phragmites australis; TN = Trapa natans). Plots show significant differences (p < 0.05) in pairwise comparison performed via Dunn’s post-hoc test with Benjamini–Hochberg adjustment. Figure S2. Comparison of NDSI correlations (p < 0.01, Bonferroni adjusted) with ETRmax for all samples (N = 324), in the visible to near-infrared spectral range (400–1000 nm): computed using Pearson’s r and Spearman’s ρ on raw data (top row), or Pearson’s r based on raw and transformed ($1/\sqrt{{ETR}_{max}}$1/ETRmax) data (bottom row). Figure S3. Comparison of NDSI correlations (p < 0.01, Bonferroni adjusted) with LMA for all samples (N = 152), in the full spectral range (400–2500 nm): computed using Pearson’s r and Spearman’s ρ on raw data (top row), or Pearson’s r based on raw and transformed ($\sqrt{LMA}$LMA) data (bottom row). Figure S4. Statistically significant (p < 0.01, Bonferroni adjusted) NDSI correlations with leaf photophysiology parameters measured on all macrophyte species sampled (N = 324) in the visible to near-infrared spectral range (400–1000 nm). Figure S5. Statistically significant (p < 0.01, Bonferro [file 13007_2021_816_MOESM1_ESM.pdf]

Leaf reflectance can surrogate foliar economics better than physiological traits across macrophyte species

Paolo Villa[1,*], Rossano Bolpagni[1, 2], Monica Pinardi[1], Viktor R. Tóth[3,*]

[1] *Institute for Electromagnetic Sensing of the Environment, National Research Council of Italy (CNR-IREA), Milan (Italy)*

[2] *Department of Chemistry, Life Sciences and Environmental Sustainability, University of Parma, Parma (Italy)*

[3] *Balaton Limnological Research Institute, Tihany (Hungary)*

***corresponding authors:**

P. Villa: *villa.p@irea.cnr.it* (email), *+39.02-23699292* (phone)

V. R. Tóth: *toth.viktor@blki.hu* (email), *+36.87-448244* (phone)

Supplementary Material

Supplementary Table S1. Summary of *in situ* samples collected for this study.

Location					Number of leaves sampled			
**Date**	**Site**	**Lat (N)**	**Lon (E)**	**Species**	**Reflectance spectra**	**Photophysiology traits**	**Biochemistry traits (pigments)**	**Structure traits**
21/07/2016	Hídvégi	46.6524	17.1433	*Trapa*	-	-	3	3
21/07/2016	Hídvégi	46.6581	17.1231	*Trapa*	-	-	3	3
21/07/2016	Hídvégi	46.6589	17.1238	*Trapa*	-	-	3	3
21/07/2016	Hídvégi	46.6131	17.1674	*Trapa*	-	-	3	3
22/07/2016	Hídvégi	46.6045	17.1665	*Trapa*	-	-	3	3
22/07/2016	Hídvégi	46.6003	17.1594	*Trapa*	-	-	3	3
22/07/2016	Hídvégi	46.6077	17.1428	*Trapa*	-	-	3	3
22/07/2016	Hídvégi	46.6135	17.1406	*Trapa*	-	-	2	2
21/07/2016	Hídvégi	46.6152	17.1675	*Nuphar*	-	-	3	3
21/07/2016	Hídvégi	46.6134	17.1676	*Nymphaea*	-	-	3	3
21/07/2016	Hídvégi	46.6146	17.1673	*Nymphaea*	-	-	3	3
27/07/2016	Mantua	45.1578	10.7463	*Nelumbo*	6	6	-	-
27/07/2016	Mantua	45.1608	10.7345	*Nuphar*	6	6	-	-
27/07/2016	Mantua	45.1601	10.7294	*Phragmites*	6	6	-	-
27/07/2016	Mantua	45.1608	10.7357	*Trapa*	5	5	-	-
27/07/2016	Mantua	45.1611	10.7331	*Trapa*	6	6	-	-
28/07/2016	Mantua	45.1624	10.7100	*Ludwigia*	-	6	-	-
28/07/2016	Mantua	45.1578	10.7141	*Phragmites*	-	6	-	-
29/07/2016	Mantua	45.1709	10.7971	*Ludwigia*	-	3	-	-
29/07/2016	Mantua	45.1643	10.8040	*Trapa*	6	6	-	-
29/07/2016	Mantua	45.1685	10.7929	*Trapa*	6	6	-	-

29/07/2016	Mantua	45.1513	10.8121	*Trapa*	6	6	-	-
29/05/2017	Mantua	45.1624	10.7101	*Ludwigia*	-	12	9	9
29/05/2017	Mantua	45.1631	10.7819	*Nelumbo*	3	3	-	-
29/05/2017	Mantua	45.1629	10.7769	*Nelumbo*	3	3	-	-
29/05/2017	Mantua	45.1604	10.7670	*Nelumbo*	3	3	-	-
29/05/2017	Mantua	45.1625	10.7092	*Nuphar*	-	11	11	12
29/05/2017	Mantua	45.1608	10.7346	*Nuphar*	4	4	4	4
29/05/2017	Mantua	45.1689	10.7869	*Nuphar*	4	4	-	-
29/05/2017	Mantua	45.1606	10.7358	*Trapa*	4	4	-	-
30/05/2017	Mantua	45.1705	10.7929	*Nymphaea*	4	4	4	4
30/05/2017	Mantua	45.1647	10.8059	*Trapa*	4	4	4	4
30/05/2017	Mantua	45.1705	10.7929	*Trapa*	4	4	4	4
30/05/2017	Mantua	45.1686	10.7926	*Trapa*	4	4	4	4
30/05/2017	Mantua	45.1490	10.8146	*Trapa*	6	6	4	4
14/06/2017	Hídvégi	46.6040	17.1667	*Trapa*	6	6	-	-
14/06/2017	Hídvégi	46.6523	17.1427	*Trapa*	6	6	-	-
14/06/2017	Hídvégi	46.6584	17.1231	*Trapa*	6	6	-	-
14/06/2017	Hídvégi	46.6124	17.1414	*Trapa*	6	6	-	-
27/07/2017	Mantua	45.1627	10.7092	*Ludwigia*	7	9	7	9
27/07/2017	Mantua	45.1633	10.7828	*Nelumbo*	6	9	9	9
27/07/2017	Mantua	45.1623	10.7739	*Nelumbo*	6	6	6	6
27/07/2017	Mantua	45.1611	10.7682	*Nelumbo*	5	6	6	6
27/07/2017	Mantua	45.1627	10.7092	*Nuphar*	3	3	2	3
27/07/2017	Mantua	45.1633	10.7473	*Trapa*	9	9	7	8
28/07/2017	Mantua	45.1704	10.7921	*Nuphar*	6	5	6	6
28/07/2017	Mantua	45.1705	10.7927	*Nymphaea*	6	6	6	6
28/07/2017	Mantua	45.1688	10.7913	*Trapa*	6	6	6	6
28/07/2017	Mantua	45.1652	10.8053	*Trapa*	6	6	6	
28/07/2017	Mantua	45.1495	10.8142	*Trapa*	6	6	5	6
17/07/2018	Hídvégi	46.6149	17.1675	*Nuphar*	6	6	-	-
17/07/2018	Hídvégi	46.6146	17.1674	*Nymphaea*	6	6	-	-
17/07/2018	Hídvégi	46.6524	17.1431	*Trapa*	5	6	-	-
17/07/2018	Hídvégi	46.6588	17.1241	*Trapa*	5	6	-	-
18/07/2018	Hídvégi	46.6003	17.1601	*Trapa*	6	6	-	-
18/07/2018	Hídvégi	46.6127	17.1413	*Trapa*	6	6	-	-
24/07/2018	Varese	45.8065	8.7661	*Ludwigia*	12	12	-	-
24/07/2018	Varese	45.8068	8.7717	*Ludwigia*	12	12	-	-
24/07/2018	Varese	45.8144	8.7581	*Nelumbo*	6	6	-	-
24/07/2018	Varese	45.8138	8.7588	*Nelumbo*	6	6	-	-
24/07/2018	Varese	45.8042	8.7756	*Trapa*	6	6	-	-
25/07/2018	Mantua	45.1626	10.7092	*Ludwigia*	10	12	12	12
25/07/2018	Mantua	45.1626	10.7098	*Ludwigia*	12	12	11	12
25/07/2018	Mantua	45.1633	10.7838	*Nelumbo*	6	6	6	6
25/07/2018	Mantua	45.1614	10.7689	*Nelumbo*	5	6	6	6
25/07/2018	Mantua	45.1632	10.7475	*Trapa*	6	6	5	6
26/07/2018	Mantua	45.1704	10.7933	*Nuphar*	6	6	6	6
26/07/2018	Mantua	45.1704	10.7933	*Nymphaea*	6	6	6	6
26/07/2018	Mantua	45.1652	10.8050	*Trapa*	6	6	6	6
26/07/2018	Mantua	45.1686	10.7920	*Trapa*	6	6	6	6
26/07/2018	Mantua	45.1482	10.8148	*Trapa*	6	6	6	6

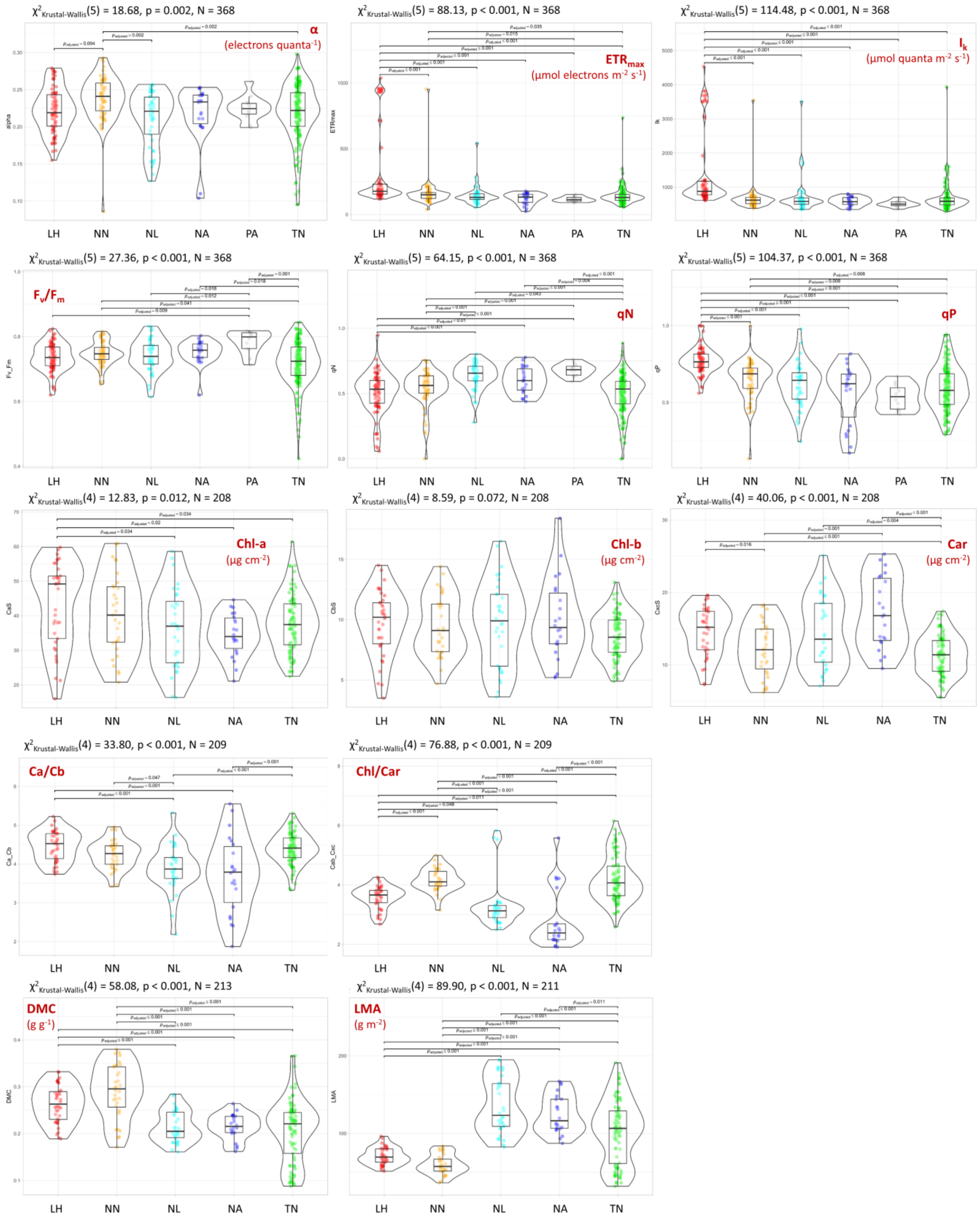

Supplementary Figure S1. Violin plots (with encompassed box plots) showing range and distribution of all leaf traits - photophysiological parameters, biochemistry (pigments) and leaf structure traits - measured over 6 macrophyte species (LH=*Ludwigia hexapetala*; NN=*Nelumbo nucifera*; NL=*Nuphar lutea*; NA=*Nymphaea alba*; PA=*Phragmites australis*; TN=*Trapa natans*). Plots show significant differences ($p < 0.05$) in pairwise comparison performed via Dunn's post-hoc test with Benjamini-Hochberg adjustment.

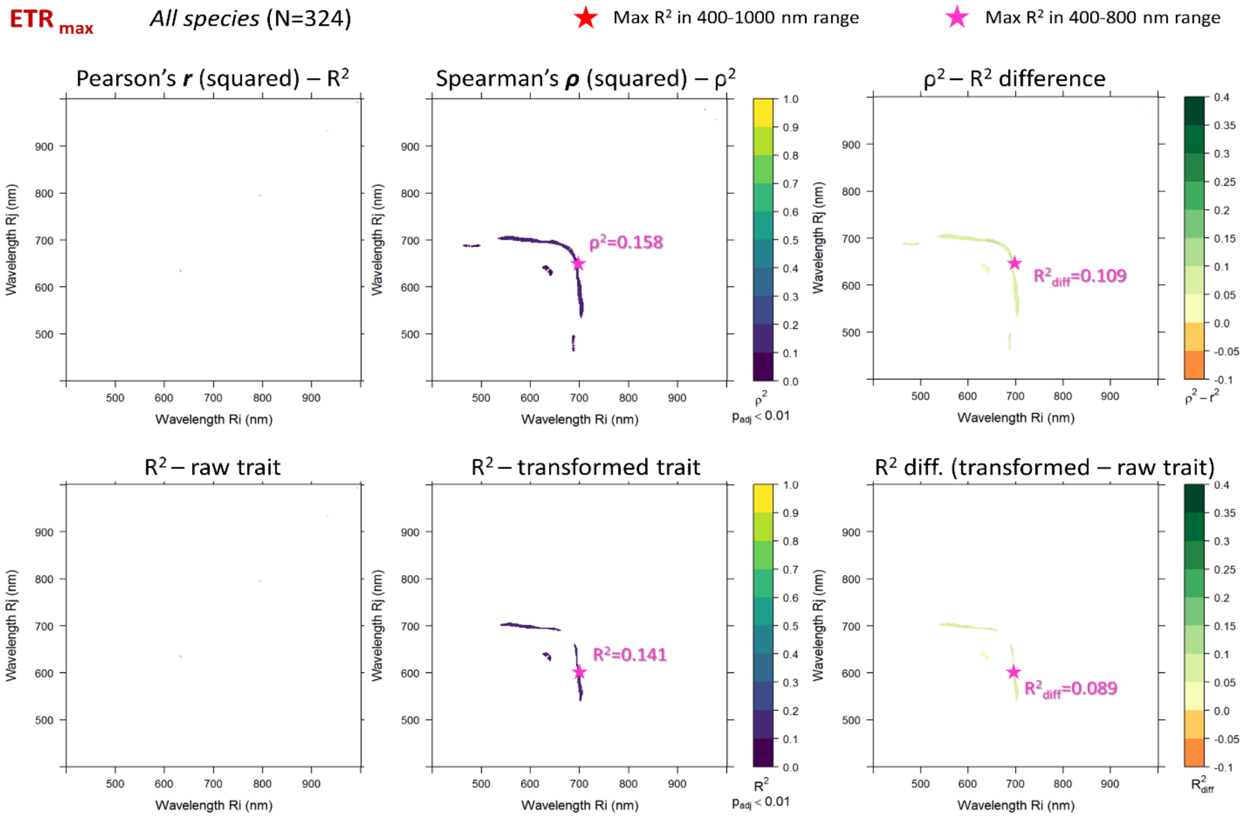

Supplementary Figure S2. Comparison of NDSI correlations ($p < 0.01$, Bonferroni adjusted) with ETR_{max} for all samples (N = 324), in the visible to near-infrared spectral range (400-1000 nm): computed using Pearson's *r* and Spearman's ρ on raw data (top row), or Pearson's *r* based on raw and transformed ($1/\sqrt{ETR_{max}}$) data (bottom row).

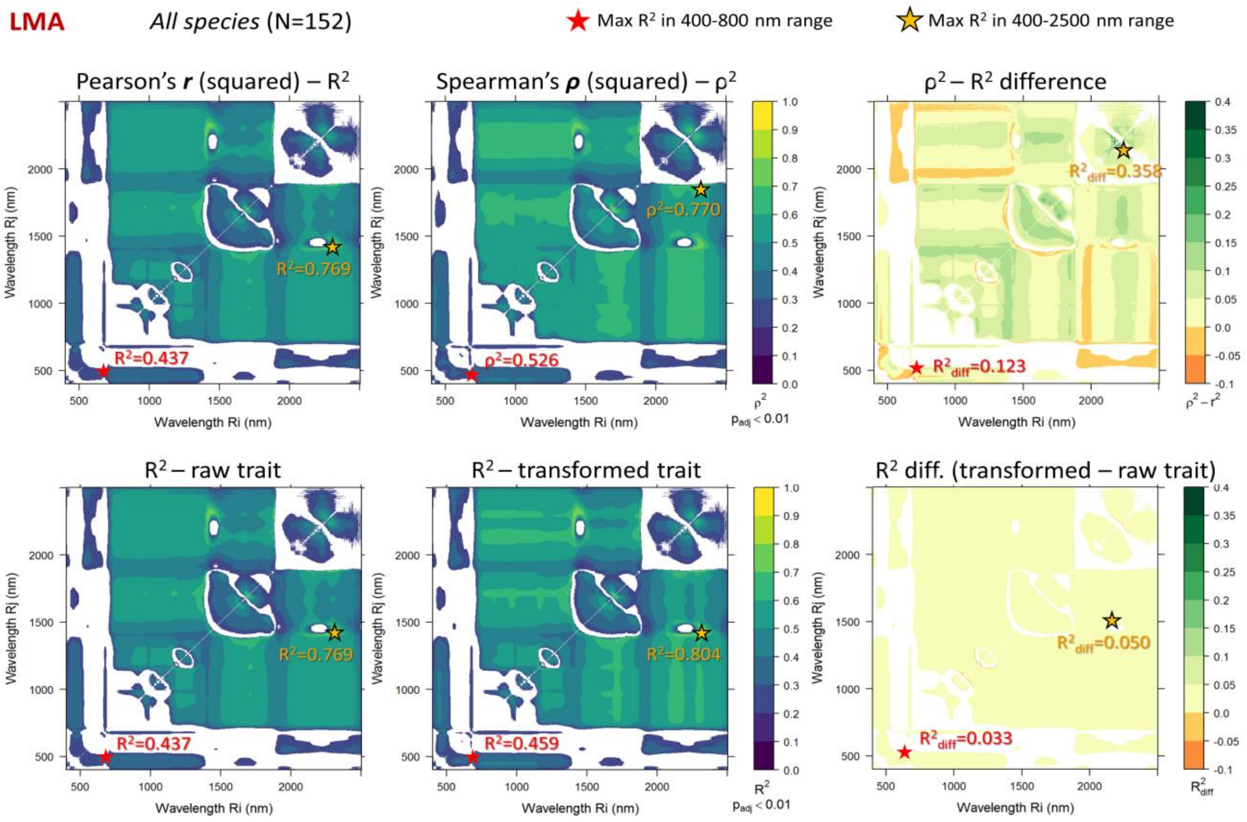

Supplementary Figure S3. Comparison of NDSI correlations ($p < 0.01$, Bonferroni adjusted) with LMA for all samples (N = 152), in the full spectral range (400-2500 nm): computed using Pearson's *r* and Spearman's ρ on raw data (top row), or Pearson's *r* based on raw and transformed ($\sqrt{LMA}$) data (bottom row).

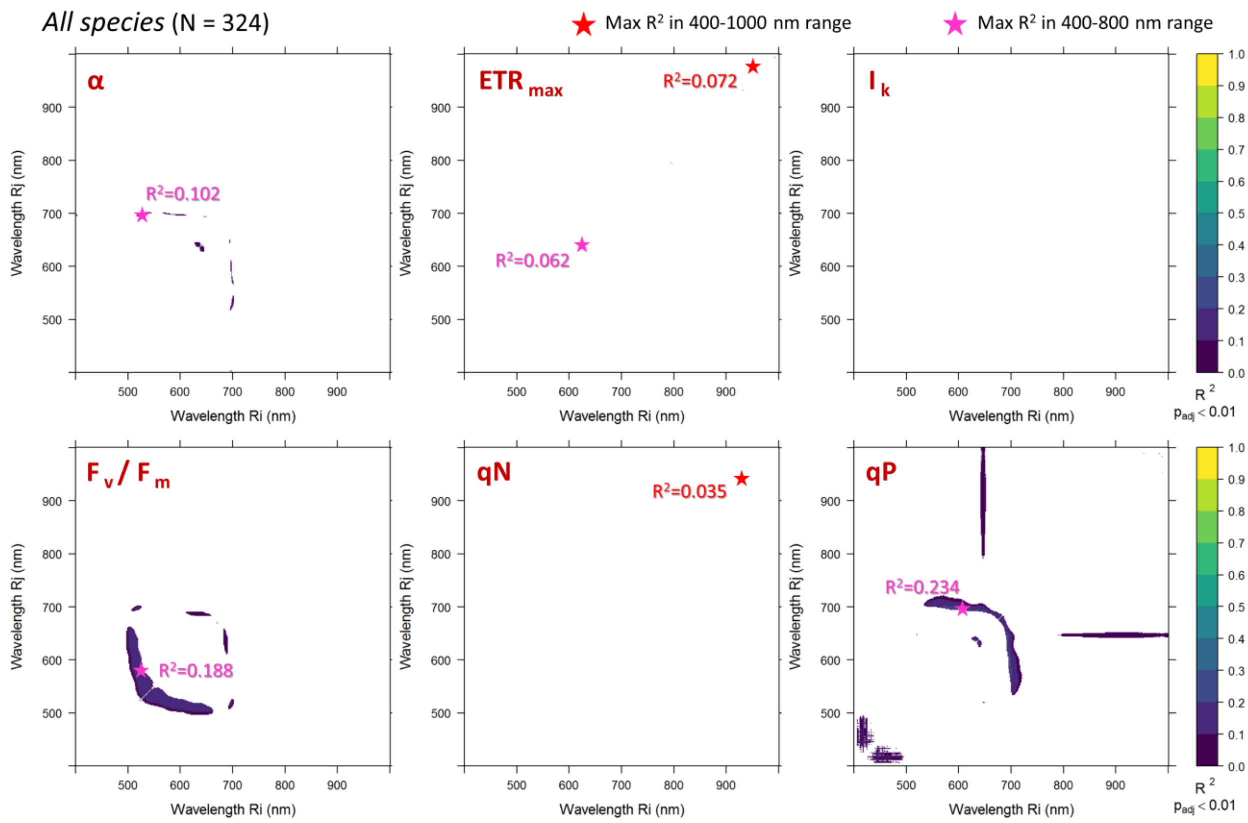

Supplementary Figure S4. Statistically significant ($p < 0.01$, Bonferroni adjusted) NDSI correlations with leaf photophysiology parameters measured on all macrophyte species sampled (N = 324) in the visible to near-infrared spectral range (400-1000 nm).

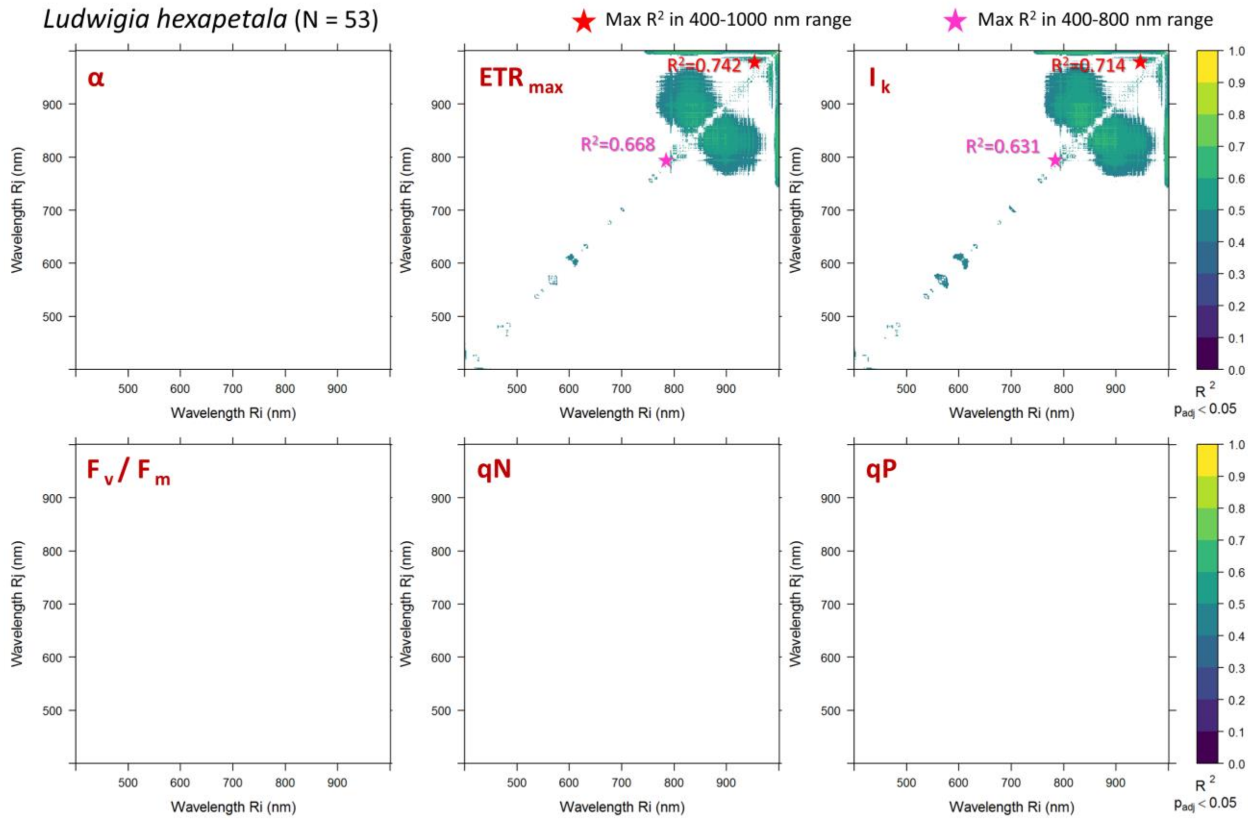

Supplementary Figure S5. Statistically significant ($p < 0.01$, Bonferroni adjusted) NDSI correlations with leaf photophysiology parameters measured on *Ludwigia hexapetala* samples (N = 53) in the visible to near-infrared spectral range (400-1000 nm).

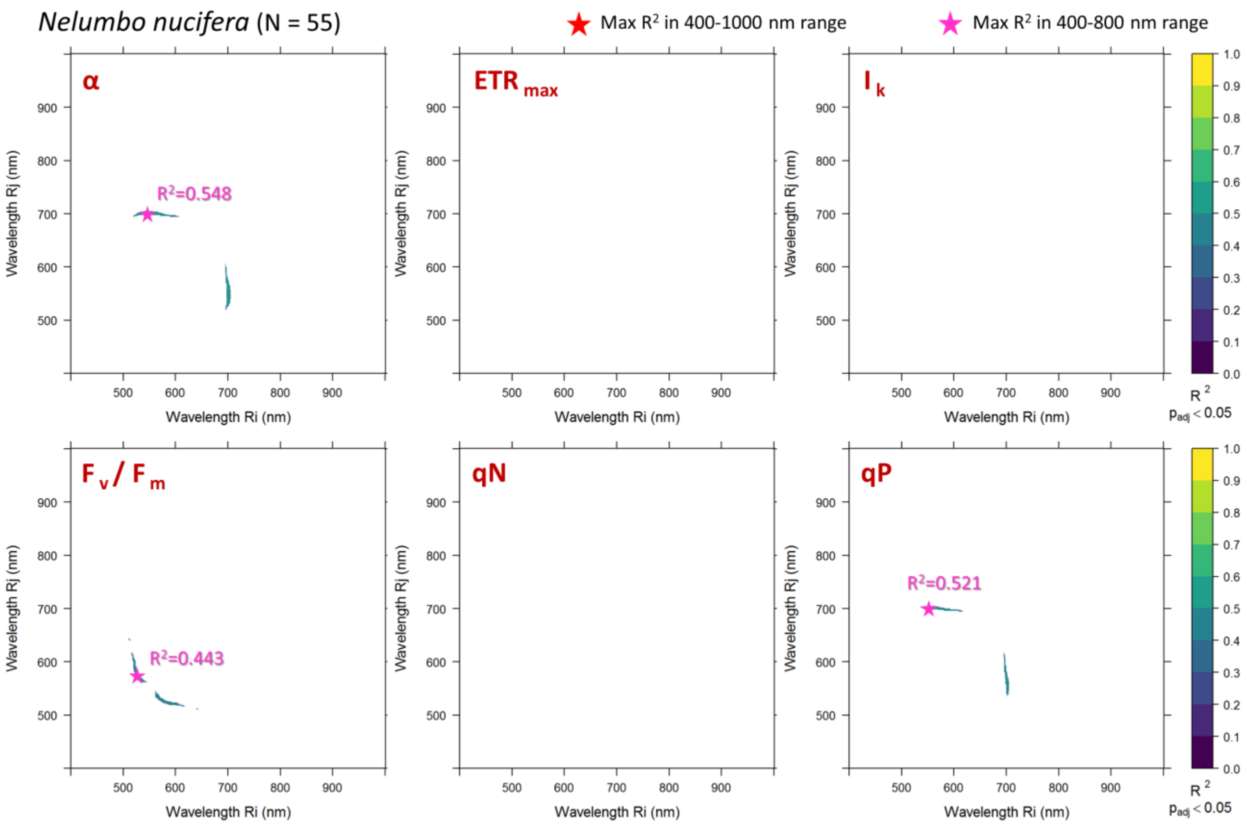

Supplementary Figure S6. Statistically significant ($p < 0.01$, Bonferroni adjusted) NDSI correlations with leaf photophysiology parameters measured on *Nelumbo nucifera* samples (N = 55) in the visible to near-infrared spectral range (400-1000 nm).

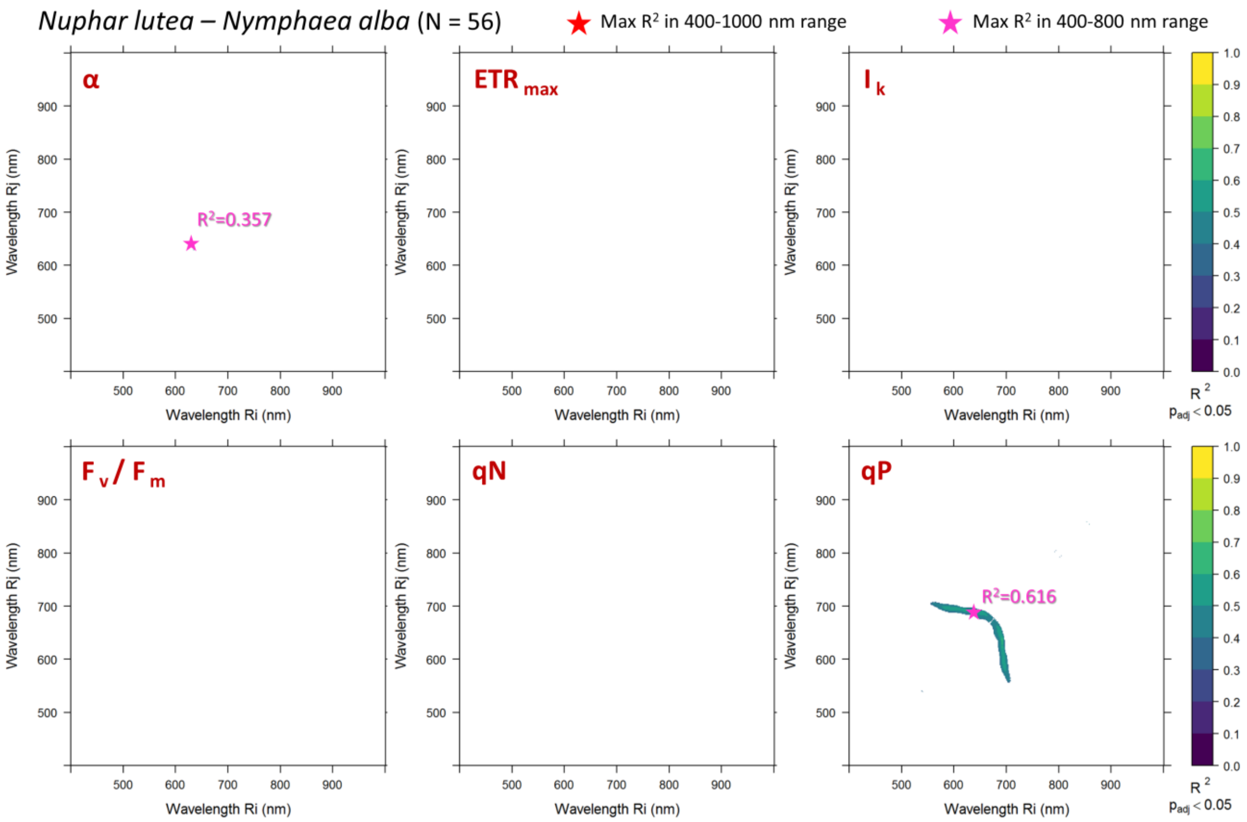

Supplementary Figure S7. Statistically significant ($p < 0.01$, Bonferroni adjusted) NDSI correlations with leaf photophysiology parameters measured on *Nuphar lutea* and *Nymphaea alba* samples (N = 57) in the visible to near-infrared spectral range (400-1000 nm).

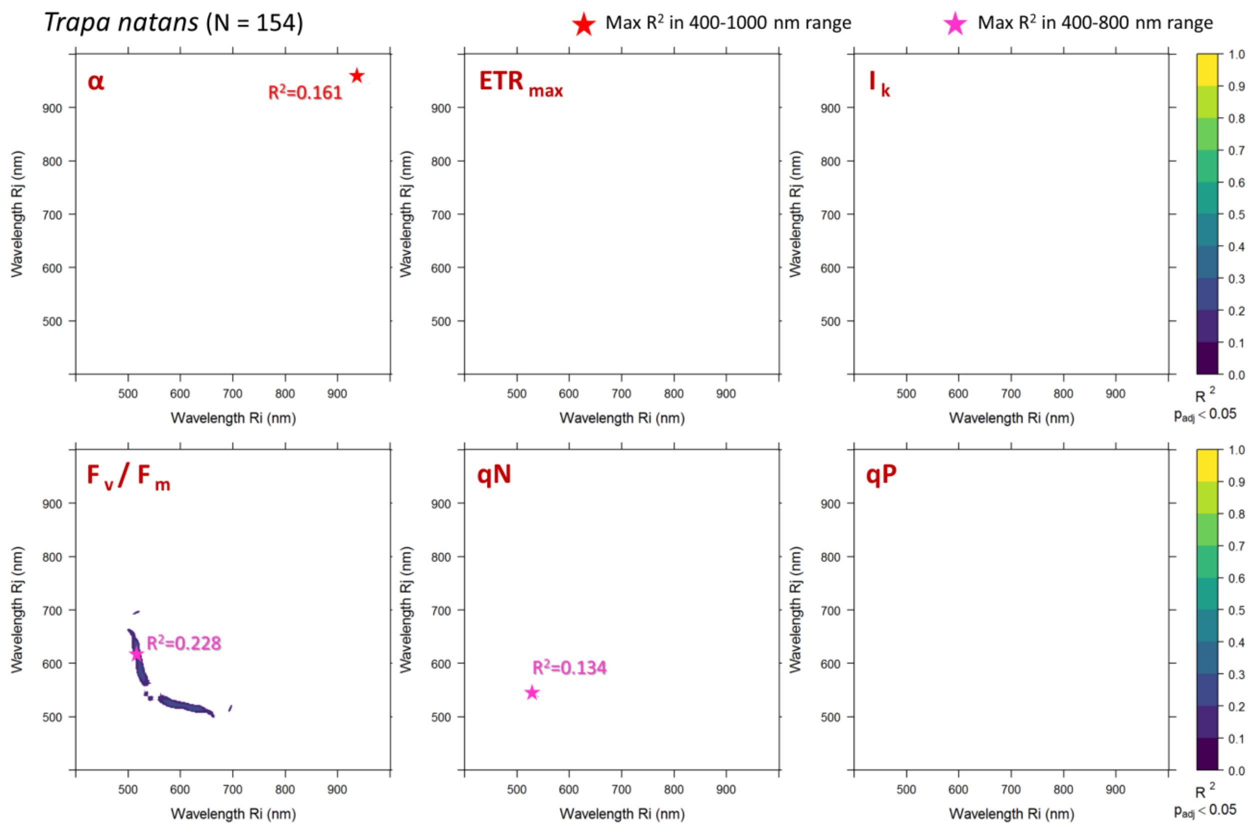

Supplementary Figure S8. Statistically significant ($p < 0.01$, Bonferroni adjusted) NDSI correlations with leaf photophysiology parameters measured on *Trapa natans* samples (N = 154) in the visible to near-infrared spectral range (400-1000 nm).

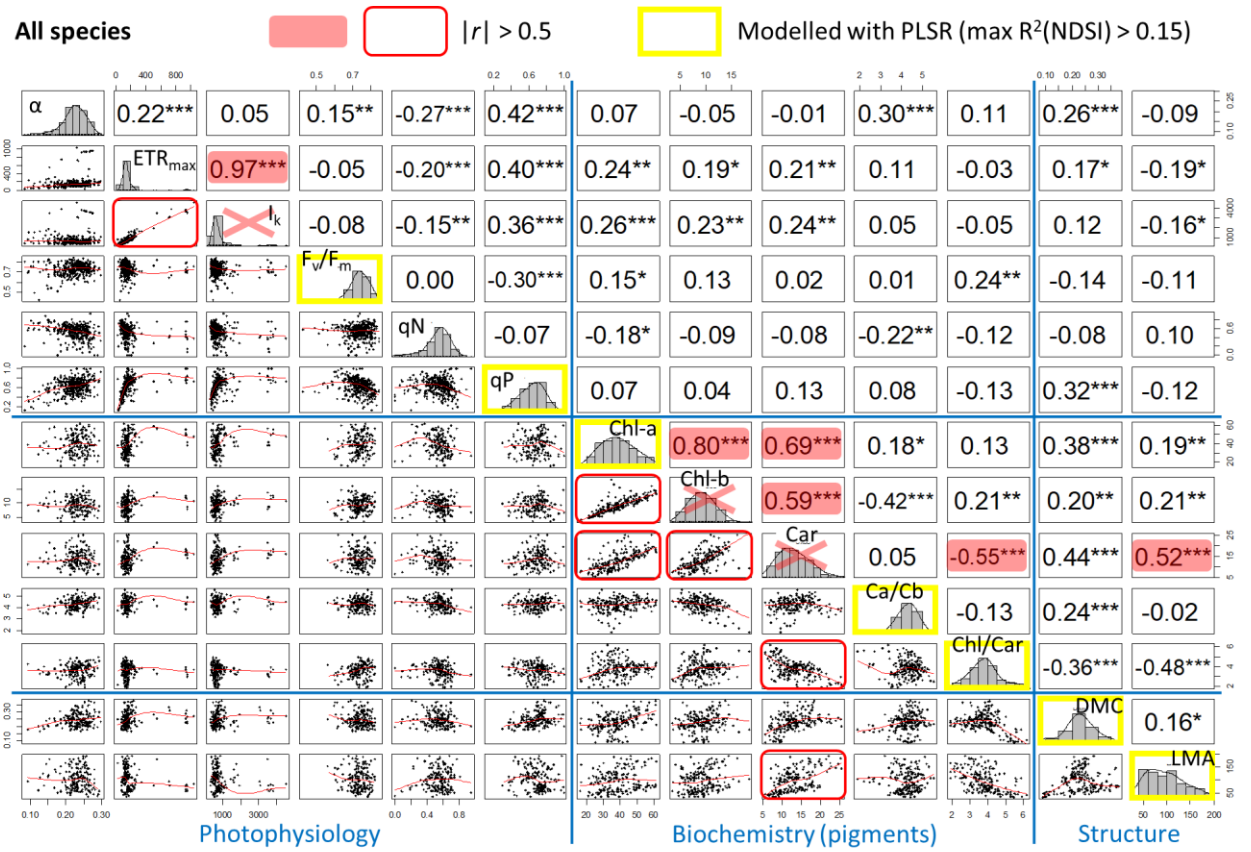

Supplementary Figure S9. Correlation matrix of leaf traits measured over 6 species. Pairwise scatter plots are shown in the lower left half, histograms are shown on the diagonal, and the coefficient of correlation (Pearson's r) of each pair of traits is shown in the upper right half, including info about its significance level (* p<0.05, ** p<0.01, *** p<0.001).

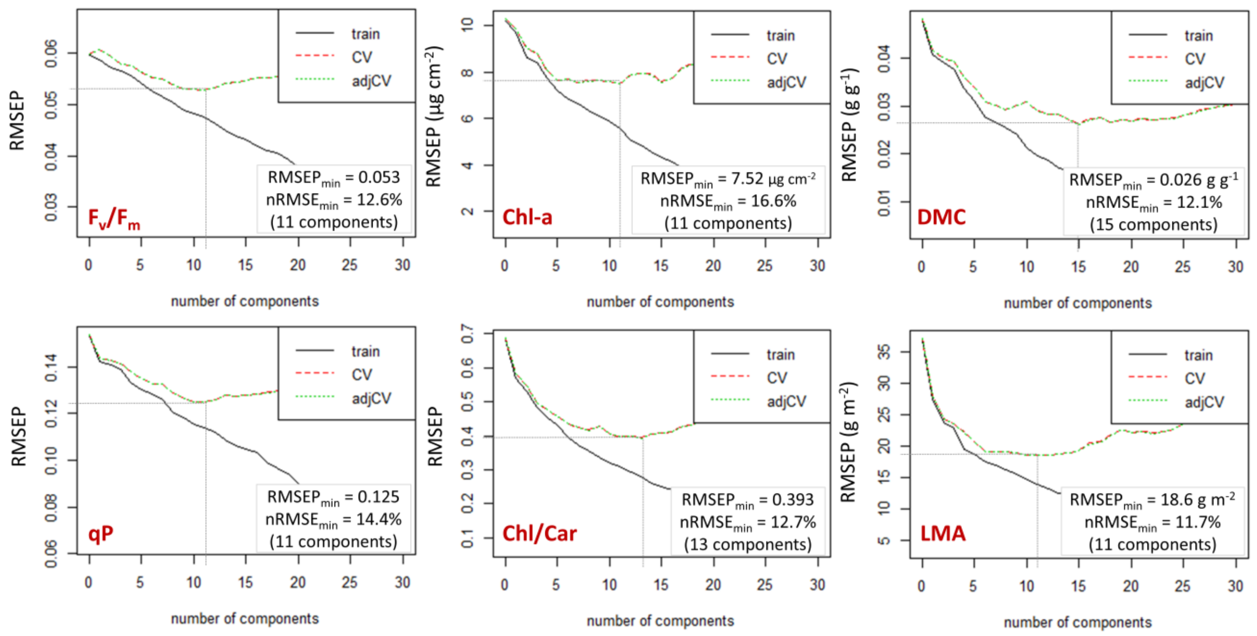

Supplementary Figure S10. Variation of root mean square error of prediction (RMSEP) with PLSR model components, computed against the full dataset (training or leave-one-out cross-validation) for selected leaf traits estimated from leaf spectral reflectance for all macrophyte species sampled.

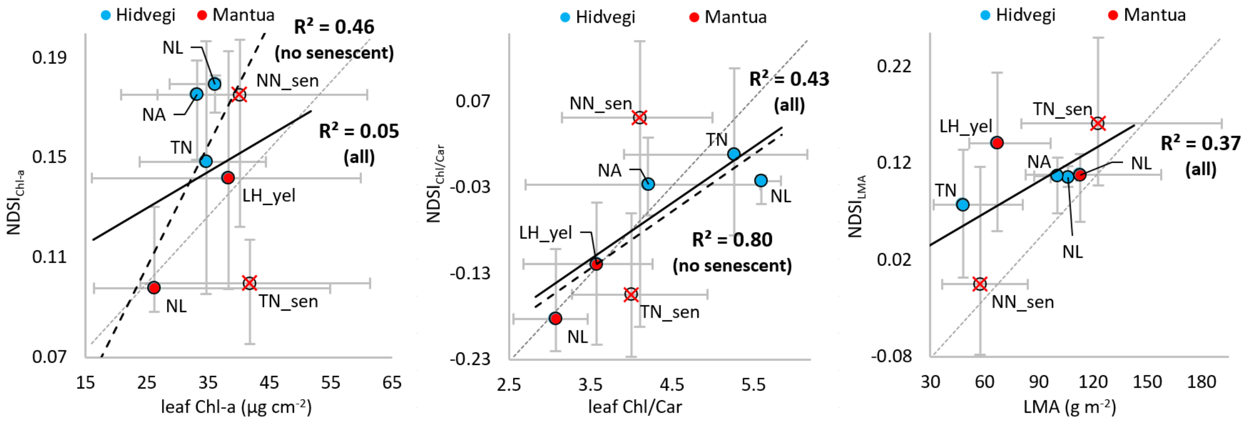

Supplementary Figure S11. Comparison of distribution of selected spectral proxies (NDSI) derived from APEX data at Lake Hídvégi and Mantua lakes system for mono-specific stands and connected leaf traits measured in our dataset (at peak of growth conditions). Data points represent median scores and whiskers delimit the extremes of values observed. Linear regression lines and their coefficient of determination (R^2) separating stands in senescence phase at the time of overflight over Mantua site (27 September 2014) are superimposed on the graphs. LH_yel: *Ludwigia hexapetala* (with signs of chlorotic conditions); NN_sen: *Nelumbo nucifera* (in early senescence phase); NL: *Nuphar lutea*; NA: *Nymphaea alba*; TN: *Trapa natans*; TN_sen: *Trapa natans* (around senescence conditions).
